# Salvianolic Acid B and Ginsenoside Re Synergistically Protect Against Ox-LDL-Induced Endothelial Apoptosis Through the Antioxidative and Antiinflammatory Mechanisms

**DOI:** 10.3389/fphar.2018.00662

**Published:** 2018-06-20

**Authors:** Ke Yang, Yun Luo, Shan Lu, Ruifeng Hu, Yuyang Du, Ping Liao, Guibo Sun, Xiaobo Sun

**Affiliations:** ^1^Beijing Key Laboratory of Innovative Drug Discovery of Traditional Chinese Medicine (Natural Medicine) and Translational Medicine, Institute of Medicinal Plant Development, Peking Union Medical College and Chinese Academy of Medical Sciences, Beijing, China; ^2^Zhongguancun Open Laboratory of the Research and Development of Natural Medicine and Health Products, Beijing, China; ^3^Key Laboratory of Bioactive Substances and Resources Utilization of Chinese Herbal Medicine, Ministry of Education, Beijing, China; ^4^Key Laboratory of Efficacy Evaluation of Chinese Medicine against Glycolipid Metabolic Disorders, State Administration of Traditional Chinese Medicine, Beijing, China; ^5^Department of Cardiovascular Medicine, The Hospital of Ningxiang County People, Changsha, China

**Keywords:** endothelial cell, salvianolic acid B, ginsenoside Re, response surface methodology, network pharmacology

## Abstract

Salvianolic acid B (SalB) and ginsenoside Re (Re) protect endotheliocytes against apoptosis through different mechanisms. However, whether both compounds could synergistically protect endothelial cells against oxidized low-density lipoprotein (Ox-LDL)-induced apoptosis is unclear. This study aimed to assess the protective effect of combined SalB and Re (SR) treatment on Ox-LDL-induced endothelial apoptosis and to explore the mechanism underlying this effect. Results showed that SalB, Re, or SR could protect against Ox-LDL-induced endothelial apoptosis. Furthermore, the composition of SR was optimized through central composite design with response surface methodology. SR with a composition of 60 μg/mL of SalB and 120 μg/mL of Re exerted the optimal protective effect. Network pharmacology research revealed that SalB and Re in SR synergistically protect against Ox-LDL-induced endothelial apoptosis by regulating oxidative stress and phlogistic pathways. *In vitro* experiments confirmed these results. Compared with the same dose of SalB or Re alone, SR significantly decreased the contents of inflammatory mediators and increased the activities of antioxidant enzymes. SR could synergistically restore the balanced redox state of the cells and inhibit the activation of nuclear transcription factor kappa B and the caspase cascade by activating the phosphatidylinositol 3 kinase/protein kinase B pathway and inhibiting the phosphorylation of p38 mitogen-activated protein kinase. These pathways are regulated by down-regulating the expression of lectin-like Ox-LDL receptor-1 and NADPH oxidase and up-regulating the expression of estrogen receptor alpha. Therefore, SR effectively prevents Ox-LDL-induced endothelial apoptosis through antioxidative and antiinflammatory mechanisms.

## Introduction

Atherosclerosis (AS), the main pathological basis of coronary artery disease and stroke, is a chronic inflammatory and multifactorial disease (Ross, 1999; Peters et al., 2012). The apoptosis of endothelial cells initiates and promotes the development of atherosclerosis (Asai et al., 2000; Chen et al., 2005; Hansson, 2005). Therefore, treatment with compounds that protect endothelial cells against apoptosis is an effective strategy for preventing and treating atherosclerosis. Among the many factors that induce endothelial apoptosis, oxidized low-density lipoprotein (Ox-LDL) is the most important (Martinet and Kockx, 2001; Chen et al., 2007). Ox-LDL first binds to lectin-like Ox-LDL receptor-1 (LOX-1) and then activates NADPH oxidase to produce numerous types of reactive oxygen species (ROS) that can initiate the caspase cascade(Chen et al., 2004); activate the p38 mitogen-activated protein kinase (p38-MAPK)-mediated signaling pathway, or inhibit the phosphatidylinositol 3 kinase/protein kinase B (PI3K/Akt) pathway. These effects accelerate the activation of nuclear transcription factor kappa B (NF-κB) and up-regulate the expression of inflammatory mediators, thus further inducing endothelial apoptosis and apoptosis-related protein expression (Li and Mehta, 2009; Lee et al., 2010; Ahsan et al., 2015). Therefore, Ox-LDL-induced endothelial apoptosis should be effectively controlled through treatment with a combination of compounds with antioxidant and antiinflammatory activities.

The commercially available Guanxin Danshen formulation which is composed mainly of *Salviae miltiorrhizae* Bunge and *Panax notoginseng* (Burkill) F. H. Chen and is widely used to treat atherosclerotic cardiovascular diseases. The detailed mechanism underlying the pharmaceutical activity of *S. miltiorrhizae* Bunge and *P. notoginseng* (Burkill) F. H. Chen has never been thoroughly elucidated. Nevertheless, their components undoubtedly exert their curative effects through multiple compounds and targets (Han et al., 2008; Li et al., 2016). The main active ingredients formula in these two herbs possess the characteristic of formulae and simplify the research of complex formulae. Therefore, the active components formula is a requisite cut-point for developing and explicating the traditional chinese medicine (Li et al., 2010; Isgut et al., 2017). Salvianolic acid B (SalB) is the highest amount of the hydrophilic phenolic acid from *S. miltiorrhizae* Bunge, and ginsenoside Re (Re) is one of the major water-soluble triol ginsenoside derived from *P. notoginseng* (Burkill) F. H. Chen. SalB exhibits antiapoptotic, antioxidant, and antiinflammatory activities (Lee et al., 2014; Ren et al., 2016; Sun et al., 2016). Meanwhile, Re exerts pharmacological effects on the cardiovascular system (Peng et al., 2012). Furthermore, evidence suggests that combining SalB or Re with other active components promotes the pharmacological activities of these compounds (Li et al., 2008; Zhou et al., 2016). However, reports on the effect of SalB or Re on Ox-LDL-induced endothelial apoptosis remain limited, and the synergistic effects of SalB and Re against Ox-LDL-induced endothelial apoptosis remain unknown.

This study aimed to test and verify the effect of SalB or Re alone on Ox-LDL-induced endothelial apoptosis and to identify the combination of SalB and Re (SR) with the optimal protective effect through response surface methodology (RSM). The dose–effect curves of the combined drug treatments were assessed through the median-effect method. Moreover, the mechanism underlying the pharmaceutical action of SR was analyzed through network pharmacology and validated *in vitro*.

## Materials and Methods

### Reagent and Materials

SalB and Re (purity > 98%) were purchased from Shanghai Winherb Medical S&T Development (Shanghai, China). Dimethyl sulfoxide (DMSO) and collagenase I were acquired from Sigma-Aldrich (St. Louis, MO, United States). All cell-culture materials were obtained from Lifeline (Randallstown, MD, United States). Ox-LDL was supplied by Union-BioTechnology (Beijing, China). Fluorescent dye (JC-1) and MTT were purchased from Enzo Life Sciences (Plymouth, PA, United States). AnnexinV/Propidium Iodide (PI) assay kit was obtained from Invitrogen (Eugene, OR, United States). Image-iT^TM^ LIVE Green Reactive Oxygen Species Detection Kit was supplied by Invitrogen (Carlsbad, CA, United States). GSH-Px, LDH, SOD, and CAT kits and Coomassie Protein Assay Kit were purchased from the Nanjing Jiancheng Institute of Biological Engineering (Nanjing, China). Caspase-3 Fluorometric Assay Kits were from BioVision (Milpitas, CA, United States). Protease Inhibitor Cocktail, Enhanced Chemiluminescence Western Blot Detection Kits, and BCA Protein Assay Kit were acquired from CWbiotech (Beijing, China). Specific kinase inhibitors, such as LY294002 (CID: 3973), were procured from Calbiochem (San Diego, CA, United States). ICI182780 (NO: S7409) was obtained from Selleckchem (Houston, TX, United States). Antibodies were obtained from Santa Cruz Biotechnology (Santa Cruz, CA, United States), Abcam (Cambridge, United Kingdom), or Cell Signaling Technology Inc. (Danvers, MA, United States). Other chemicals were purchased from Sigma (St. Louis, MO, United States).

### Preparation and Cultivation of Cells

According to previously described (Sun et al., 2013), HUVECs were isolated from fresh human umbilical veins by using 0.1% collagenase I. After 13 min enzymatic dissociation, HUVECs were collected by centrifugation at 3600 rpm for 15 min and cultured on 1% gelatin-coated plastic dishes by VascuLife^®^ Medium (CID: LL0003) supplemented with 1% penicillin/streptomycin (v/v). The cells were cultured in a humidified incubator with 95% air and 5% CO_2_ at 37°C. Cells from passages 3–7 were used for subsequent experiments, and media were refreshed every 2 days. All experiments using HUVEC cells were approved by the Ethics Committee of Peking Union Medical College (SYXK-2013-0023, Beijing, China) and was administrated in accordance with the Declaration of Helsinki. The neonate cords were donated by the Maternal and Child Care Service Centre in Beijing, China. The study protocol was explained and all participating donors were given written informed consents.

### Assay for Cell Viability

To detect the protective effects of SalB or Re dissolved in DMSO on HUVECs, HUVECs were cultured in 96-well plates at a density of 10^4^ cells/well and grown for 24 h. Then, the HUVECs were pretreated with a concentration gradient of SalB (3.125, 6.25, 12.5, 25, 50, 100 μg/mL) or Re (6.25, 12.5, 25, 50, 100, 200 μg/mL) for 12 h in serum-free medium. Next, the HUVECs were incubated with 100 μg/mL of Ox-LDL for 24 h which did not have SalB or Re. Subsequently, 5 mg/mL of MTT in fresh medium was added to each well, and the HUVECs were incubated for an additional 4 h. Finally, 150 μL DMSO was added to dissolve the formazan crystals after removing the supernatant. Absorbance was measured by a plate reader (Infinite M1000, Tecan, Sunrise, Austria) at 570 nm.

### The 2^k^ Factor Design Experiment

The two factors were investigated: SalB; Re. The influence of variations in these factors on the cell viability was evaluated using a 2^k^ factorial design (**Table 1**). A total of four experimental runs were conducted (randomly) for which three replicates were performed. According to the result of the single-factor experiment, the concentration range of SalB is 50 – 100 μg/mL and Re is 70 – 140 μg/mL. This gave the same conditions in each experiment. The outputs of the experimental design were analyzed using Design Expert 8.0.6 software (Stat-Ease, Inc.). The magnitude and direction of factor effect were determined via OD calculations and the significance of the factor effect was evaluated using ANOVA (**Table 2**).

**Table 1:** 2^k^ factorial experiment: Salvianolic acid B and ginsenoside Re dosage.

SalB (μg/mL)	Re (μg/mL)	OD
50	140	0.68112
100	70	0.59444
50	70	0.63527
100	140	0.65002

**Table 2:** Climbing test: Salvianolic acid B and ginsenoside Re dosage.

Run	Scheme	SalB (μg/mL)	Re (μg/mL)	OD
1	Zero point (0)	75	105	0.704735
2	Step (Δ)	-4	5	
3	0 + 1 Δ	71	110	0.788305
4	0 + 2 Δ	67	115	0.81443
**5**	**0 + 3** Δ	**63**	**120**	**0.8356225**
6	0 + 4 Δ	59	125	0.7690275
7	0 + 5 Δ	55	130	0.7972225
8	0 + 6 Δ	51	135	0.7771925

*Bold values mean that this combination is best*

### Steepest Ascent Test

In order to determine the suitable ranges which tend to approach the optimal condition, the steepest ascent test was designed based on significant factors. The path began at the center point of the 2^k^ factorial design, serving as the origin for the steepest ascent experiment, that’s mean that the initial concentration of SalB is 75 μg/mL and Re is 105 μg/mL. The direction of steepest ascent was decided by the first-order mathematical linear equation predicted from the 2^k^ factorial design, so the direction of SalB was –4 and the Re was +5 (**Table 2**). Four runs were evaluated over one plate with replicated three, the same conditions were given in each experiment. Finally, the cell viability was evaluated. Statistical analysis and model prediction were performed using Design Expert 8.0.6 software (Stat-Ease, Inc.).

### Response Surface Methodology

Response surface methodology with central composite design (CCD) was performed to optimize the combination of SalB and Re (SR). A two-factor, five-level center CCD was finished. CCD was created by Design Expert 8.0.6 software (Stat-Ease, Inc.), and thirteen combinations, including six replicates at central point was chosen randomly according to CCD which are listed in **Table 3**. Based on the result of the steepest ascent test, the initial concentration of SalB is 63 μg/mL and Re is 120 μg/mL. The data analysis, model construction, and 3D response surface graph generation were performed using Design Expert 8.0.6 software (Stat-Ease, Inc.).

**Table 3:** Optimization of compatibility of salvianolic acid B and ginsenoside Re by response surface methodology.

Run	SalB (μg/mL)	Re (μg/mL)	OD
1	46	100	0.654798
2	80	100	0.674065
3	46	140	0.792487
4	80	140	0.671063
5	39	120	0.71337
6	87	120	0.665192
7	63	92	0.657268
8	63	148	0.680782
9	63	120	0.80618
10	63	120	0.79084
11	63	120	0.78349
12	63	120	0.80414
13	63	120	0.8143

### Analysis of the Median-Effect Equation

To further research SalB–Re interactions in SR, a range of SR was analyzed through the median-effect method. According to the previous description (Chou, 2006), experiment scheme of SR was designed by CalcuSyn 2.1 (Cambridge, United Kingdom), and six combinations listed in **Table 4** were evaluated over one plate with replicated three, the same conditions were given in each experiment. Finally, SalB–Re interactions in SR were assessed on the basis of the CI value derived by CalcuSyn 2.1 (Cambridge, United Kingdom) and were defined as follows: CI > 1 antagonistic, CI = 1 additive, and CI < 1 synergistic.

**Table 4:** Evaluation of interaction between salvianolic acid B and ginsenoside Re by medium efficiency principle.

SalB (μg/mL)		Re (μg/mL)		SalB:Re	1:2
Dose	Effect	Dose	Effect	Dose	Effect
10	0.3399475	20	0.34045	10	0.6768475
20	0.455375	40	0.456825	20	0.743375
30	0.5746225	60	0.5885125	30	0.8105175
40	0.6177875	80	0.650175	40	0.89841
50	0.66165175	100	0.70795	50	0.929985
60	0.7272075	120	0.78258	60	0.983965

### Validation With Cell Models

To further verify the protective effects of SalB, Re, or SR, other two models were tested using H9c2 cells obtained from the Chinese Academy of Sciences Cell Bank (Shanghai, China). The first model was H_2_O_2_-induced apoptosis of H9c2 cardiomyoblasts (Sun et al., 2011), in a nutshell, H9c2 cells were cultured on high glucose DMEM supplemented with supplemented with 1% penicillin/streptomycin (v/v). The cells were cultured in a humidified incubator with 95% air and 5% CO_2_ at 37°C for 24 h. When cells reached 70 – 80% confluence, cells were incubated with 60 μg/mL of SalB, 120 μg/mL of Re and SR (60 μg/mL – 120 μg/mL) for 12 h, and then removed the supernatant. Next, these cells were incubated with 150 mM H_2_O_2_ for 6 h. At last cell viability was measured by MTT.

The second model was hypoxia–reoxygenation-induced apoptosis of H9c2 (Liao et al., 2016). Briefly, the cells were cultured on high glucose DMEM supplemented with supplemented with 1% penicillin/streptomycin (v/v). The cells were cultured in a humidified incubator with 95% air and 5% CO_2_ at 37°C for 24 h. When cells reached 70 – 80% confluence, cells were incubated with 60 μg/mL of SalB, 120 μg/mL of Re and SR (60 μg/mL – 120 μg/mL) for 12 h, and then removed the supernatant. Next, these cells were cultured in 37°C for 6 h in an anaerobic glove box, where normal air was removed by a combination of 5% CO_2_, 5% H_2_ and 90% N_2_, and then they were removed to the regular incubator for 12 h with the medium replaced by high glucose medium to mimic reperfusion. At last cell viability was measured by MTT. In addition, all experiments were accomplished on the HUVECs except for the part of validation with cell models, which used H9c2 cells to prove the effect of SR.

### Construction of Pathways or Disease Networks Targeted by SalB and Re

First, information on SalB and Re targets were first collected from the literature (Web of Science, Pubmed, ScienceDirect, SpringerLink, CNKI). The cut-off date for entries is June 17, 2016. Second, changes in gene expression following the Ox-LDL activation of LOX-1 in endothelial cells was studied by analyzing GSE13139. Third, the Ox-LDL-induced endothelial apoptosis database was constructed with a protein chip (Qin et al., 2015). Furthermore, atherosclerosis-related genes were retrieved from GeneCards^1^. Finally, molecular docking was performed by idTarget^2^ and PharmMapper^3^. The framework for the target genes, pathways, or disease networks of SalB and Re was thus generated.

Gene expression profiles were analyzed using the bioconductor packet. DAVID 6.7 was used for GO enrichment analysis^4^. KEGG pathways were analyzed using Kobas 2.0.^5^

A network was constructed to better understand the complex relationships between SalB and Re, their target pathways and associated diseases. The network was structured and analyzed using Cytoscape 3.4.0. The interactions of target genes were analyzed using STRING^6^.

### Flow Cytometric Detection of Apoptosis

Early apoptosis and necrosis were measured using the Annexin V-FITC Apoptosis Detection Kit. Briefly, HUVECs were treated with or without the concentrations of 60 μg/mL of SalB, 120 μg/mL of Re and SR (60 μg/mL – 120 μg/mL) for 12 h, followed by 100 μg/mL Ox-LDL for another 24, and then the treated cells were harvested and washed twice with cold PBS. Subsequently, 10^6^ cells were incubated with 10 μL of Annexin-V-FITC and 1 μL of PI working solution (100 μg/mL) in the dark for 15 min at 37°C. Finally, cell culture plates were filled with 500 μL of binding buffer. Cellular fluorescence was measured through flow cytometry (BD, San Jose, CA, United States).

### Measurement of Mitochondrial Membrane Potential

The change in mitochondrial membrane potential was detected through JC-1 staining. HUVECs were pre-incubated with or without the concentrations of 60 μg/mL of SalB, 120 μg/mL of Re and SR (60 μg/mL – 120 μg/mL) for 12 h, followed by 100 μg/mL Ox-LDL for another 24, and then the treated cells were incubated with JC-1 (10 μM final concentration) at 37°C in the dark for 30 min. Next, the treated cells were washed three times with warm PBS, harvested and dissolved in 500 μL PBS. Finally, the intensity of green fluorescence was determined at an excitation wavelength of 485 nm and an emission wavelength of 535 nm. The intensity of red fluorescence was determined at an excitation wavelength of 585 nm and an emission wavelength of 590 nm. The fluorescence intensity of the cell was analyzed by using a microplate reader (Infinite M1000, Tecan).

### Measurement of LDH Levels and CAT, GSH-Px, and SOD Activities

HUVECs were cultured at 10^4^ cells/well in 6 – well plates for 24 h. The cells were pre-incubated with or without the concentrations of 60 μg/mL of SalB, 120 μg/mL of Re and SR (60 μg/mL – 120 μg/mL) for 12 h, followed by 100 μg/mL Ox-LDL for another 24, and then the supernatant was collected for the following experiments. The activities of GSH-Px, SOD, and CAT, and the release of LDH were quantified using commercial kits in accordance with the manufacturer’s instructions.

### Analysis of ROS Activity

HUVECs were cultured at 10^4^ cells/well in 6 – well plates for 24 h. The cells were pre-incubated with or without the concentrations of 60 μg/mL of SalB, 120 μg/mL of Re and SR (60 μg/mL – 120 μg/mL) for 12 h, followed by 100 μg/mL Ox-LDL for another 24, and then the supernatant was removed. After treatment, cells were collected and incubated with carboxy-H2DCFDA (25 μM) in the dark for 30 min at 37°C, and then washed thrice with PBS, and quantitatively analyzed through flow cytometry (BD, San Jose, CA, United States).

### Determination of Inflammatory Mediator Contents

HUVECs were cultured at 10^4^ cells/well in 6 – well plates for 24 h. The cells were pre-incubated with or without the concentrations of 60 μg/mL of SalB, 120 μg/mL of Re and SR (60 μg/mL – 120 μg/mL) for 12 h, followed by 100 μg/mL ox-LDL for another 24, and then the supernatant was collected for the following experiments. Standard ELISA kits were used to measure IL-6, TNF-α, and MCP-1 contents in accordance with the manufacturer’s instructions.

### Analysis of Caspase-3 Activation

Caspase-3 activation was measured using a fluorescein-active caspase-3 staining kit in accordance with the manufacturer’s instructions. Briefly, HUVECs were cultured at 10^4^ cells/well in 6 – well plates for 24 h. The cells were pre-incubated with or without the concentrations of 60 μg/mL of SalB, 120 μg/mL of Re and SR (60 μg/mL – 120 μg/mL) for 12 h, followed by 100 μg/mL Ox-LDL for another 24, and the supernatant was removed. Then, 10^6^ cells were harvested and incubated with 1 ml of the substrate FITC-DEVD-FMK in the dark for 1 h at 37°C. Subsequently, the cells were centrifuged for 5 min at 3,000 rpm, and the supernatant was removed. Finally, the cells were washed twice with PBS and resuspended in 300 ml of wash buffer on ice. The samples were tested through flow cytometry (BD, San Jose, CA, United States).

### Western Blot Analysis of Protein Expression

Protein expression was analyzed through Western blot analysis. In short, HUVECs were cultured at 3 × 10^4^ cells/well in 25 cm^2^ culture plates for 24 h. The cells were pre-incubated with the concentrations of 60 μg/mL of SalB, 120 μg/mL of Re and SR (60 μg/mL – 120 μg/mL) for 12 h, with or without anisomycin (1 μM) or LY294002 (10 nM) for another 1 h. Then, the cells were incubated with 100 μg/mL Ox-LDL for another 24, and the supernatant was removed. After treatment, HUVECs were harvested and dissolved with cell lysis buffer containing 1% protease inhibitor cocktail. The lysate was centrifuged for 20 min at 12,000 *g* and 4°C to remove insoluble materials. Finally, total cellular protein was extracted as previously described (Yang et al., 2017), and each sample (30 μg of protein) was separated by SDS–PAGE and transblotted onto PVDF membranes. Next, the membranes were blocked with 5% skim milk for 2 h. Finally, the membranes were incubated with primary and secondary antibodies. The following primary antibodies were used: β-actin (C-2): sc-8432; LOX-1: 17958-1-AP; NOX4: ab109225; ERα (HC-20): sc-543; p38MAPK: #9926; p-p38MAPK: #9910; p-Akt1/2/3 (Ser 473): sc-101629; Akt1/2/3 (H-136): sc-8312; NF-κB: sc-8008; Bcl-2 (N-19): sc-492; Bax (N-20): sc-493; Smac: ab32023; and cIAP2: ab32059. The membranes were visualized using ECL chemiluminescence detection system (Bio-Rad, United States). At least three independent experiments were conducted.

### Statistical Analysis

All experiments were repeated at least thrice. All quantitative data were presented as mean ± standard deviation and analyzed through Student’s *t*-test or ANOVA by SPSS (version 20.0, Chicago, IL, United States). *P* < 0.05 was considered significant.

## Results

### Effect of Single Factors on the Protective Effects of SR Against Ox-LDL-Induced Endothelial Injury

**FIGURE 1:**
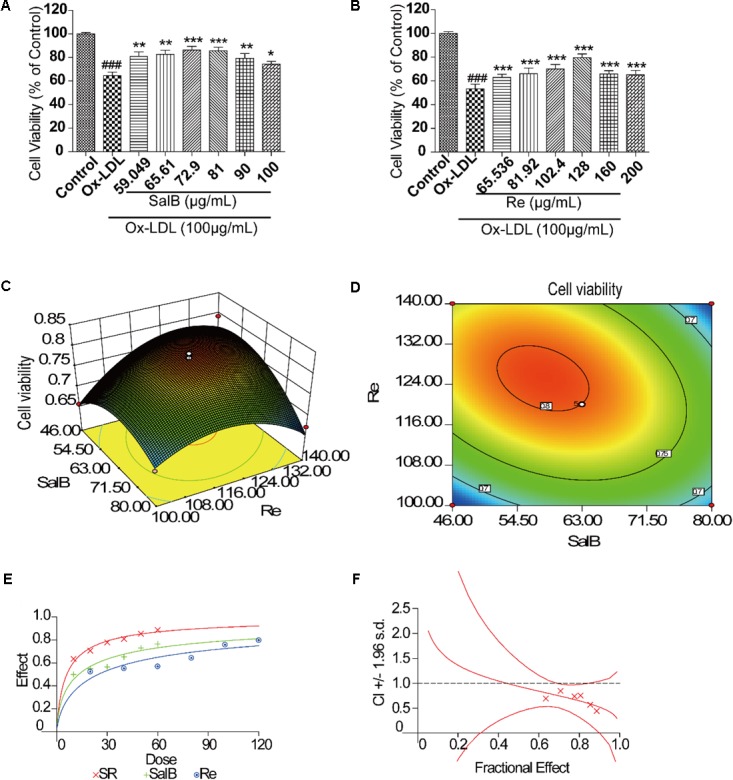
Flow path of formulation building and optimization by RSM. **(A)** Incubation with SalB for 12 h significantly reduced Ox-LDL-induced cell injury. **(B)** Incubation with Re for 12 h significantly reduced Ox-LDL-induced cell injury. **(C)** Three-dimensional graphic surface representing the effects of SalB and Re on cell viability. **(D)** Two-dimensional graphic surface representing the effects of SalB and Re on cell viability. **(E)** Dose-effect curve of SR. **(F)** Combination index of SR.^###^*P* < 0.001 vs. Control; ^∗^*P* < 0.05, ^∗∗^*P* < 0.01, ^∗∗∗^*P* < 0.001 vs. Ox-LDL.

We assessed the protective effects of SalB or Re on endothelial cells by performing 3-(4,5-dimethylthiazol-2yl)-2,5-diphenyltetrazolium bromide (MTT) assay. We found that treatment for different durations with different doses of SalB or Re alone could attenuate Ox-LDL-induced endothelial injury (Supplementary Figures 1, 2). Furthermore, we observed the ideal protective against Ox-LDL-induced endothelial injury when we treated cells with 50 μg/mL of SalB and 100 μg/mL of Re. We identified the optimal concentration of SalB or Re by refining the concentration range of these components on the basis of the above conclusions. As shown in **Figure 1A**, 12 h of treatment with 59.05–90 μg/mL SalB or 102.4–160 μg/mL Re provided the optimal protective effect against Ox-LDL-induced endothelial injury (**Figure 1B**): 81 μg/mL SalB could make the survival rate of HUVECs reach 75.33% and 128 μg/mL Re was 83.25%. Therefore, we used 50–100 μg/mL of SalB and 70–140 μg/mL of Re in further experiments.

### Formulation Building and Optimization by RSM

After the 2^k^ factor design experiment and the steepest ascent test, we applied RSM with CCD to identify the optimal formulation of SR. The 2^k^ factor design experiment showed that the optimization direction and step length of SalB are -4 and those of Re is 5, and the initial SR formulation is 75 – 105 μg/mL (SalB – Re, SR) (Supplementary Material 2). The result of the steepest ascent test indicated that the ideal dosage of SR is 63–120 μg/mL (Supplementary Material 3). We then used CCD to optimize SR formulation, as shown in Supplementary Material 4. Thirteen sets of experimental data were analyzed by a quadratic model and subjected to analysis of variance (ANOVA). Subjecting the quadratic model to ANOVA revealed that the quadratic regression equation fitted the response surface shown in Supplementary Material 4 (*R*^2^= 0.9481, adjusted *R*^2^= 0.9111). The response surface plot of cell viability versus SalB and Re concentration is shown in **Figures 1C,D**, where SR has an optimal formulation of 57.8 μg/mL SalB and 125.2 μg/mL Re. Thus, we used the combination of 60 μg/mL of SalB and 120 μg/mL of Re in the follow-up study.

We performed the median-effect method to explore the interaction of SalB and Re. The value of the combination index (CI) is between 0 and 1, indicating that SalB and Re act cooperatively (**Figures 1E,F**). Meanwhile, statistical analysis of the quadratic regression equation also implied that the components of SalB and Re were significant interactions (Supplementary Material 4).

### Validation of the Protective Effect of SR

**FIGURE 2:**
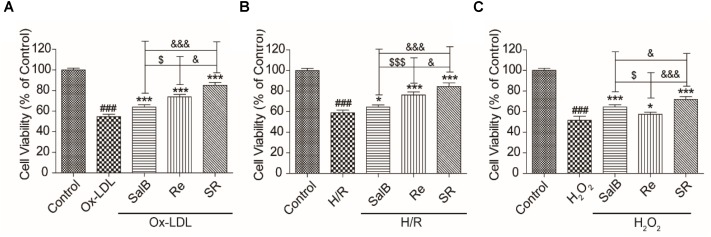
Validation of the effect of SR on Ox-LDL-induced cell injury by using different cell models. **(A)** Incubation with SalB, Re, or SR for 12 h significantly reduced Ox-LDL-induced cell injury. **(B)** Incubation with SalB, Re, or SR for 12 h significantly reduced H/R-induced H9c2 injury. **(C)** Incubation with SalB, Re, or SR for 12 h significantly reduced H_2_O_2_-induced H9c2 injury.^###^*P* < 0.001 vs. Control; ^∗^*P* < 0.05, ^∗∗^*P* < 0.01, ^∗∗∗^*P* < 0.001 vs. Model.

To test the reliability of the optimization method, we used three different cell models to confirm that SR exerts protective effects. As shown in **Figure 2**, the protective effect of 120 μg/mL of Re on Ox-LDL-induced endothelial injury and H/R-induced H9c2 injury is stronger than that of 60 μg/mL of SalB (**Figures 2A,B**). However, the effect of SalB is stronger than that of Re in the H_2_O_2_-induced H9c2 injury model (**Figure 2C**). In general, the protective effect of SR was the most efficient group in different cell models: it could make the survival rate of HUVECs reach 89.01% on Ox-LDL-induced endothelial injury, it also could make the survival rate of cells reach 84.35% in the model of H/R-induced H9c2 injury and the effectiveness of SR was 71.73% in the model of H_2_O_2_-induced H9c2 injury (**Figure 2**).

### Construction and Analysis of Networks

**FIGURE 3:**
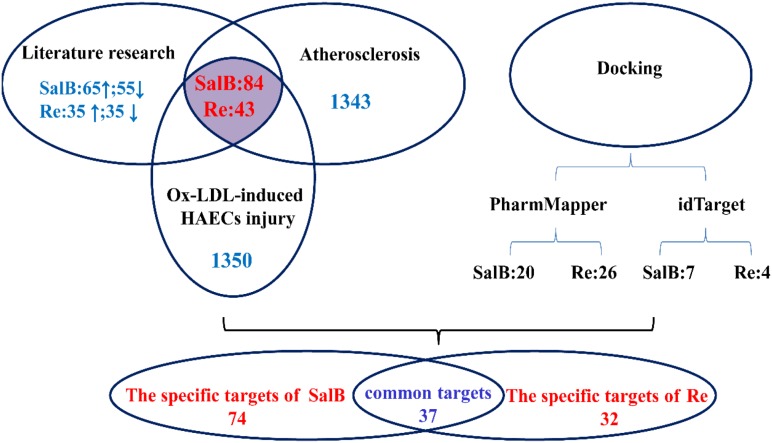
Framework of target gene identification.

To elucidate the combinatorial principle and the molecular mechanism underlying the synergetic protective effects SalB and Re against Ox-LDL-induced endothelial injury (**Figure 3**), we first designed an integrated strategy for exploring potential targets on the basis of the network pharmacology framework. We identified 111 candidate targets of SalB, of which 74 are specific targets of SalB and 37 are common targets of SalB and Re. Given that Re has 32 specific targets, we obtained 69 candidate targets of Re.

**FIGURE 4:**
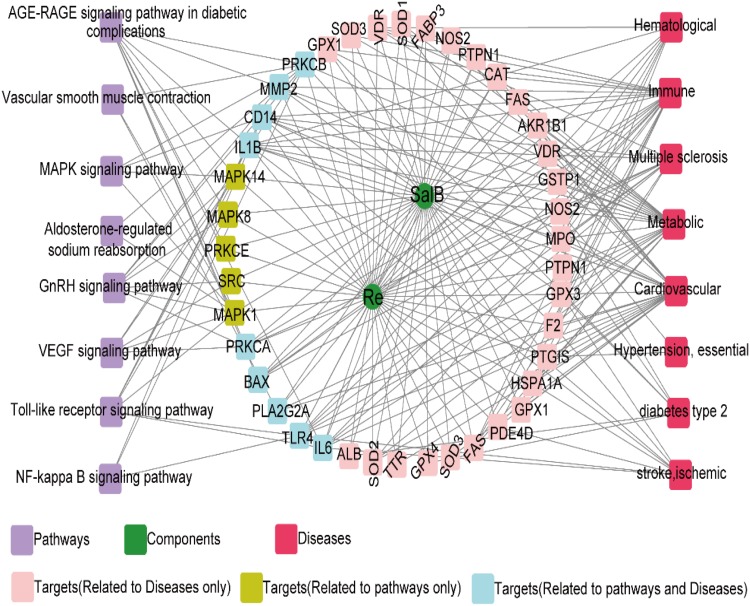
Target genes shared by SalB and Re regulate the network “SalB and Re–target genes–pathway or disease”.

To further investigate the potential pharmacological effects of SR against AS, we constructed compound–target pathways and disease networks on the basis of component–target interaction. As shown in **Figure 4**, the 37 common targets of SalB and Re mainly regulate the signaling pathways of oxidative stress, inflammation, cell proliferation, and glycolipid metabolism. The analysis of compound–target–disease networks indicated that the physiological effects of these 37 targets are mainly related to CVD or glycolipid metabolism diseases, such as metabolic disease and diabetes type II.

**FIGURE 5:**
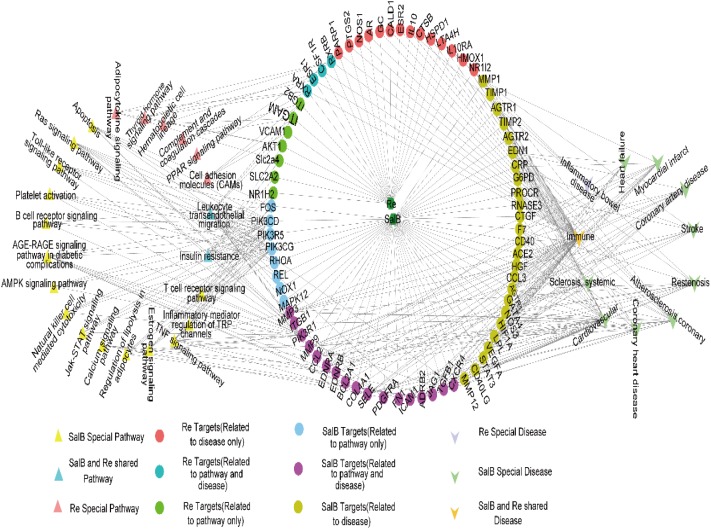
Specific target genes of SalB and Re regulate the network “SalB and Re–target genes–pathway or disease.”

We found that the specific targets of SalB mainly regulate the signal pathways of apoptosis, inflammation, and energy metabolism, whereas those of Re mainly participate in the pathways of lipid metabolism and blood coagulation. Furthermore, the signal pathways of insulin resistance and leukocyte transendothelial migration are regulated by the specific targets of SalB and Re (**Figure 5**). Next, the analysis of compound–target–disease networks revealed that the specific targets of SalB are related to cardiovascular diseases, particularly to atherosclerosis, whereas those of Re mainly regulate inflammatory bowel diseases. Most interestingly, the specific targets of SalB and Re can regulate immune diseases (**Figure 5**).

To explore the key genes regulated by SalB and Re, we analyzed gene interactions through STRING. The key genes of the common targets of SalB and Re are *SOD1, SOD2, SOD3, CAT, GPX, TLR4, SRC*, and *MAPKS* (Supplementary Figure 3). The key genes of the specific targets of SalB are *PI3K, MAPKs, MMPs, CD40, RHOA, FOS*, and *END1* (Supplementary Figure 4). The key genes of the specific targets of Re are *ESR1, ESR2*, and *PPARD* (Supplementary Figure 5).

### Effects of SR on the Apoptosis of Endothelial Cells

**FIGURE 6:**
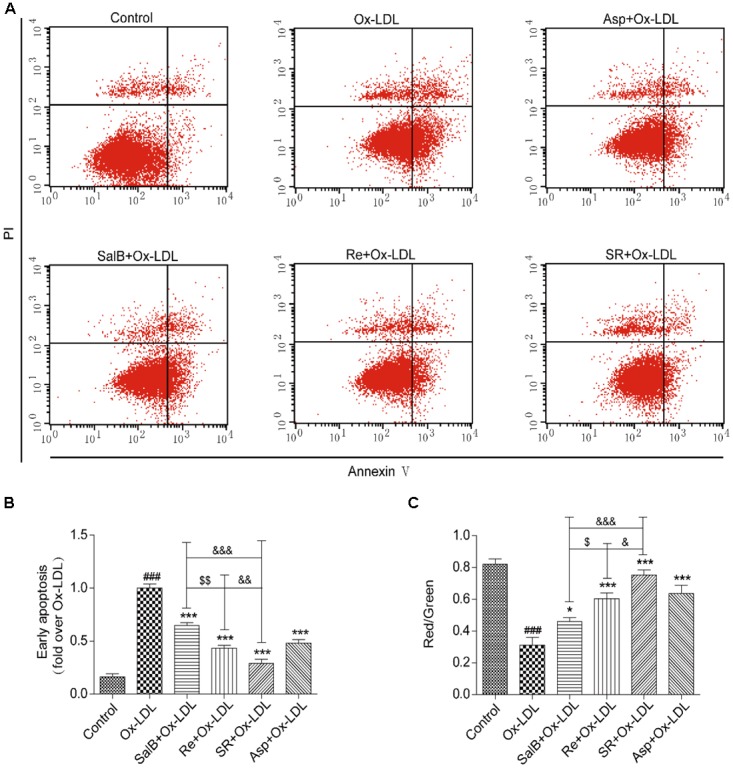
Protective effects of SalB, Re, and SR on HUVECs undergoing Ox-LDL-induced early apoptosis. **(A)** Annexin V/PI staining was used to detect the protective effects of SalB, Re, and SR on HUVECs undergoing Ox-LDL-induced apoptosis. **(B)** Quantitative analysis of the apoptosis rates of HUVECs. **(C)** JC-1 staining was used to detect mitochondrial membrane potential changes in SalB-, Re-, or SR-treated HUVECs during Ox-LDL-induced apoptosis. ^∗∗∗^*P* < 0.001, ^∗^*P* < 0.05 vs. Ox-LDL; ^###^*P* < 0.001 vs. Control; ^$$^*P* < 0.01, ^$^*P* < 0.05 vs. SalB; ^&&&^*P* < 0.001, ^&^*P* < 0.05 vs. SR.

First, we validated the effects of SR on Ox-LDL-induced endothelial apoptosis. We examined the apoptosis rates of endothelial cells by using Annexin V-FITC/PI double dyes. As shown in **Figures 6A,B**, the apoptosis rates of SalB-, Re-, or SR-treated human umbilical vein endothelial cells (HUVECs) markedly decreased relative to those of Ox-LDL-treated HUVECs. The protective effect of SR on Ox-LDL-induced apoptosis in HUVECs is the strongest among that of other monotherapies, and the viability of cells in the Re treatment group is stronger than that in the SalB treatment group (**Figures 6A,B**).

To test the antiapoptotic effect of SR, we measured the effect of SR on mitochondrial permeability, a key indicator of early apoptosis. The SR-treated group exhibited the highest red/green fluorescence intensity ratio. The red/green fluorescence intensity ratio of the Re group is lower than that of the SR group, and that of the SalB is the lowest (**Figure 6C**). Therefore, the intensity of the antiapoptotic effect exerted by the compounds follows the order of SR > Re > SalB.

### Effects of SR on the ROS Generation and Antioxidant Enzyme Activity

**FIGURE 7:**
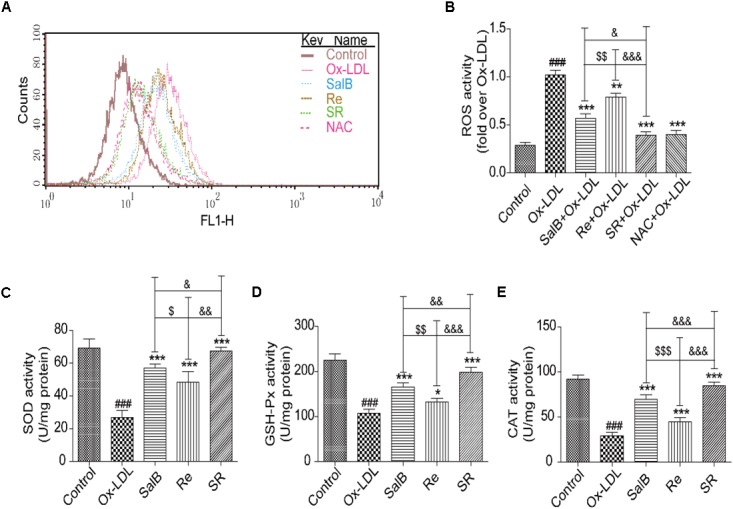
Protective effects of SalB, Re, and SR on HUVECs under Ox-LDL-induced oxidative stress. **(A,B)** Flow cytometry was performed to quantitatively analyze the protective effects of SalB, Re, and SR on the Ox-LDL-induced overexpression of ROS. **(C–E)** Commercial kits were used to determine the effects of SalB, Re, and SR on the levels of SOD, GSH-Px, and CAT activities in HUVECs undergoing Ox-LDL-induced apoptosis. ^###^*P* < 0.001 vs. Control; ^∗∗∗^*P* < 0.001, ^∗∗^*P* < 0.01, ^∗^*P* < 0.05 vs. Ox-LDL; ^$$$^*P* < 0.001, ^$$^*P* < 0.01, ^$^*P* < 0.05 vs. SalB; ^&&&^*P* < 0.001, ^&&^*P* < 0.01, ^&^*P* < 0.05 vs. SR.

Then, we validated the effects of SR on ROS generation and antioxidant enzyme activity in accordance with the results of network pharmacology. As shown in **Figures 7A,B**, incubating HUVECs with SalB, Re, or SR for 12 h prior to Ox-LDL exposure markedly reduced ROS generation by these cells. The inhibitory effect of SR is the strongest, and that of SalB is stronger than that of Re. Moreover, analyzing antioxidant enzyme activity revealed that the levels of superoxide dismutase (SOD), glutathione peroxidase (GSH-Px), and catalase (CAT) in the treated groups are significantly higher than those in the Ox-LDL group (**Figures 7C–E**). The ability of the components to regulate antioxidant enzyme activity is consistent with their ability to inhibit ROS production.

### Effects of SR on the Secretion of Adhesion Molecules and Proinflammatory Mediators

**FIGURE 8:**
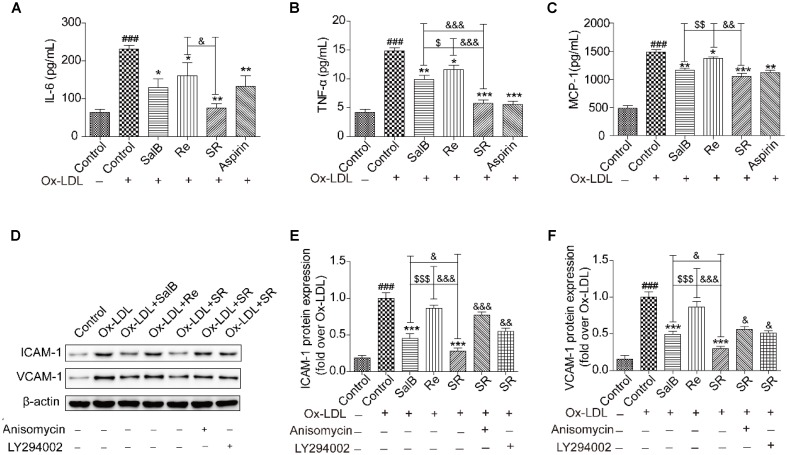
SalB, Re, and SR inhibit the Ox-LDL-induced secretion of inflammatory cytokines in HUVECs. **(A–C)** ELISA kits were used to determine the protective effects of SalB, Re, and SR on the secretion of IL-6, TNF-α, and MCP-1 in HUVECs undergoing Ox-LDL-induced apoptosis. **(D–F)** Pretreatment with anisomycin or LY294002 attenuated the protective effects of SalB, Re, and SR on the expression levels of ICAM-1 and VCAM-1 in HUVECs undergoing Ox-LDL-induced apoptosis. ^###^*P* < 0.001 vs. Control; ^∗∗∗^*P* < 0.001, ^∗∗^*P* < 0.01, ^∗^*P* < 0.05 vs. Ox-LDL; ^$$$^*P* < 0.001, ^$$^*P* < 0.01, ^$^*P* < 0.05 vs. SalB; ^&&&^*P* < 0.001, ^&&^*P* < 0.01, ^&^*P* < 0.05 vs. SR.

The results of network pharmacology showed that SR can regulate the secretion of inflammatory factors, cytokines, and adhesion molecules. We further validated the effects of SR on the activity or expression of related factors by using enzyme-linked immunosorbent assay kits (ELISA) or by performing Western blot analysis. As shown in **Figures 8A–C**, the activities of interleukin (IL)-6, monocyte chemotactic protein (MCP)-1, and tumor necrosis factor (TNF)-α are significantly up-regulated in the Ox-LDL group relative to those in the control group. However, compared with Ox-LDL treatment, SalB, Re, or SR treatment down-regulated IL-6, TNF-α, and MCP-1 expression in HUVECs undergoing Ox-LDL-induced apoptosis. SR more effectively decreased IL-6, TNF-α, and MCP-1 activities than SalB and Re, and SalB is more effective than Re.

Notably, under SalB, Re, or SR treatment, the expression patterns of ICAM-1 and VCAM-1 were different from those of IL-6, TNF-α, and MCP-1. The expression levels of ICAM-1 and VCAM-1 were more effectively inhibited by SR than by SalB and were unaffected by Re (**Figures 8D–F**).

### Mediatory Effects of SR on the Expression of LOX-1, NOX4, and ERα

**FIGURE 9:**
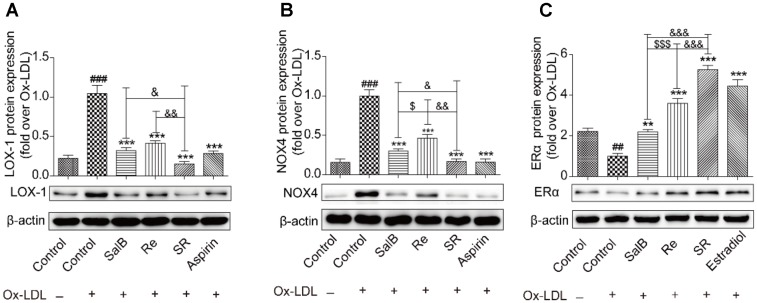
SalB, Re, and SR regulate the expression of related receptors in HUVECs undergoing Ox-LDL-induced apoptosis. **(A)** SalB, Re, and SR regulated the expression of LOX-1 in HUVECs undergoing Ox-LDL-induced apoptosis. **(B)** SalB, Re, and SR regulated the expression of NOX4 in HUVECs undergoing Ox-LDL-induced apoptosis. **(C)** SalB, Re, and SR regulated the expression of ERα in HUVECs undergoing Ox-LDL-induced apoptosis. ^###^*P* < 0.001, ^##^*P* < 0.01 vs. Control; ^∗∗∗^*P* < 0.001, ^∗∗^*P* < 0.01 vs. Ox-LDL; ^$$$^*P* < 0.001, ^$^*P* < 0.05 vs. SalB; ^&&&^*P* < 0.001, ^&&^*P* < 0.01, ^&^*P* < 0.05 vs. SR.

To study the protective mechanism of SR, we first explored the expression patterns of LOX-1, NOX4, and ERα through gene interaction analysis. As shown in **Figures 9A,B**, Ox-LDL treatment up-regulated the protein levels of LOX-1 and NOX4. As expected, pretreatment with SalB, Re, or SR could reverse this phenomenon, and SR is more effectively reversed the expression of these proteins than SalB or Re. SalB more effectively regulated NOX4 expression than Re. However, the regulatory effects of SalB and Re on LOX-1 expression did not significantly differ. In addition, SalB and Re activated ERα expression (**Figure 9C**). The effect of SR is strongest among those of the three treatments, and the effect of Re is stronger than that of SalB.

### SR Inhibits the Ox-LDL-Induced Apoptosis of HUVECs via p38MAPK and PI3K/Akt

**FIGURE 10:**
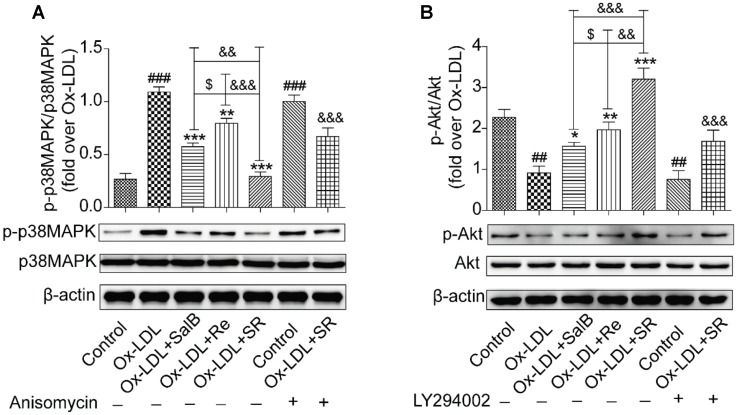
Effects of SalB, Re, and SR on PI3K/Akt and p38MAPK pathways in HUVECs undergoing Ox-LDL-induced apoptosis. **(A)** Pretreatment with anisomycin attenuated the SalB-, Re-, or SR-mediated phosphorylation of p38MAPK in HUVECs undergoing Ox-LDL-induced apoptosis. **(B)** Pretreatment with LY294002 attenuated the SalB-, Re-, or SR-mediated phosphorylation of Akt in HUVECs undergoing Ox-LDL-induced apoptosis. ^###^*P* < 0.001, ^##^*P* < 0.01 vs. Control; ^∗∗∗^*P* < 0.001, ^∗∗^*P* < 0.01, ^∗^*P* < 0.05 vs. Ox-LDL; ^$^*P* < 0.05 vs. SalB; ^&&&^*P* < 0.001, ^&&^*P* < 0.01 vs. SR.

We continued to study the downstream signaling pathways of the key gene targets of SalB and Re by analyzing receptor protein and gene expression. We found that Ox-LDL treatment could remarkably up-regulate the phosphorylation of p38MAPK compared with the control treatment. Pretreatment with SalB, Re, or SR could significantly down-regulate the Ox-LDL-induced phosphorylation of p38MAPK. SR exhibited the most intense inhibitory action among all treatments. The inhibitory action of SalB is stronger than that of the Re groups (**Figure 10A**). The addition of a p38MAPK agonist markedly reduced the protective effect of SR against Ox-LDL-induced apoptosis in HUVECs (**Figure 10A**). However, compared with the phosphorylation of p38MAPK, the effect of SalB, Re, or SR on PI3K/Akt was in the opposite direction (**Figure 10B**). Compared with the control treatment, Ox-LDL treatment could markedly down-regulate the phosphorylation level of Akt (S473). However, preincubation with SalB, Re, or SR significantly increased the phosphorylation level of Akt compared with preincubation with Ox-LDL. SR exhibited the strongest activating effect among all three treatments, and Re exhibited a stronger effect than SalB. To determine the role of the SR in PI3K/Akt activation, we examined the effects of LY294002 (12.5 nM) treatment on the activation of PI3K/Akt pathways and found that SR treatment continued to protect HUVECs against Ox-LDL-induced apoptosis (**Figure 10B**). These results indicated that SR protected against Ox-LDL-induced apoptosis in HUVECs by inhibiting p38MAPK and activating PI3K/Akt.

### Inhibitory Effects of SR on the Nuclear Translocation of NF-κB

**FIGURE 11:**
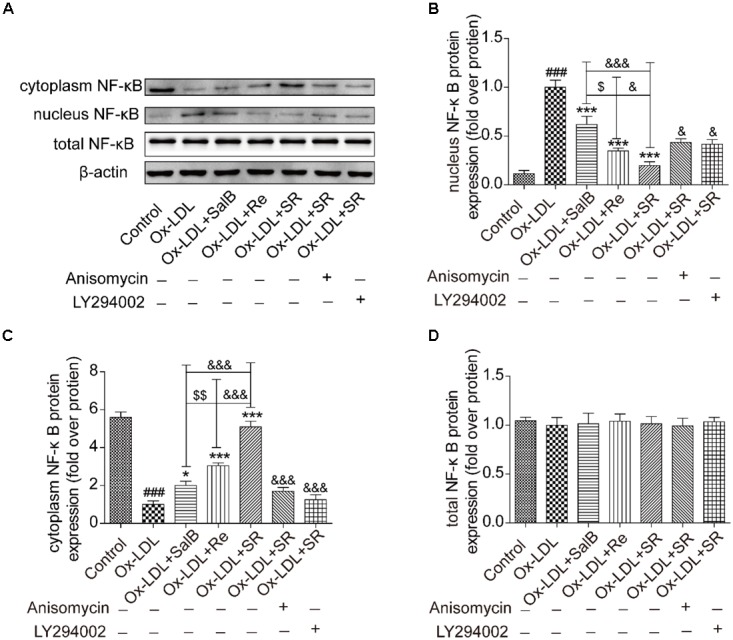
Effects of SalB, Re, and SR on the nuclear translocation of NF-κB in HUVECs undergoing Ox-LDL-induced apoptosis. **(A–D)** Anisomycin or LY294002 attenuated the SalB-, Re-, or SR-mediated nuclear translocation of NF-κB in HUVECs undergoing Ox-LDL-induced apoptosis. ^###^*P* < 0.001 vs. Control; ^∗∗∗^*P* < 0.001, ^∗^*P* < 0.05 vs. Ox-LDL; ^$$^*P* < 0.01 vs. SalB; ^&&&^*P* < 0.001, ^&&^*P* < 0.01 vs. SR.

Given that nuclear transcription factor kappa B (NF-κB) is a key downstream factor of p38MAPK and PI3K/Akt, we performed Western blot analysis to study the nuclear translocation of NF-κB. As shown in **Figure 11**, Ox-LDL treatment could markedly activate the nuclear translocation of NF-κB from the cytoplasm to the nucleus relative. However, SalB, Re, or SR pretreatment could significantly reverse this effect. The inhibitory action of SR is the strongest among those of all three treatments, and that of Re is stronger than that of SalB. However, that addition of anisomycin (an activator of p38MAPK, 1 nM) or LY294002 (an inhibitor of PI3K, 12.5 nM) weakened the inhibitory action of SR. These results indicated that SR inhibits the nuclear translocation of NF-κB through the p38MAPK and PI3K/Akt pathways.

### SR Restores the Balanced Expression of Proapoptotic and Antiapoptotic Proteins

**FIGURE 12:**
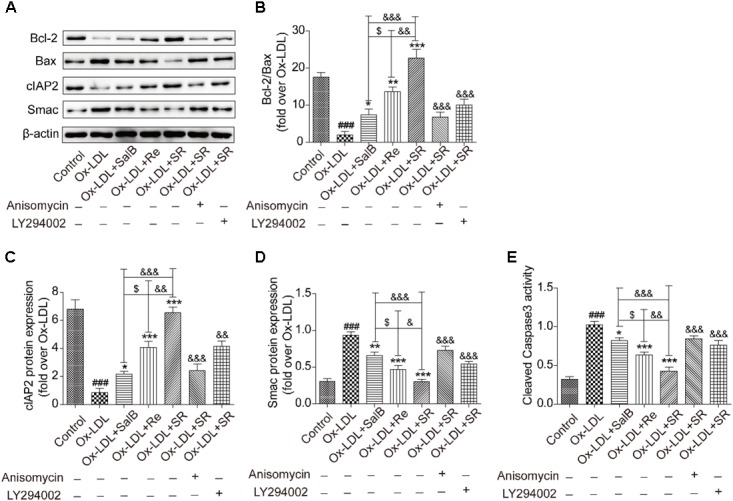
SalB, Re, and SR regulate the expression of apoptosis-related proteins in HUVECs undergoing Ox-LDL-induced apoptosis through PI3K/Akt/NF-κB and p38MAPK/NF-κB pathways. **(A–D)** Treatment with anisomycin or LY294002 attenuated the regulatory effects of SalB, Re, and SR on the expression levels of Bcl-2, Bax, cIAP2, and Smac in HUVECs undergoing Ox-LDL-induced apoptosis. **(E)** Statistical analysis of the effects of SalB, Re, and SR on the activity of cleaved caspase3 in HUVECs undergoing Ox-LDL-induced apoptosis. ^###^*P* < 0.001 vs. Control; ^∗∗∗^*P* < 0.001, ^∗∗^*P* < 0.01, ^∗^*P* < 0.05 vs. Ox-LDL; ^$^*P* < 0.05 vs. SalB; ^&&&^*P* < 0.001, ^&&^*P* < 0.01,^&^*P* < 0.05 vs. SR.

Given the important role of NF-κB in modulating cell survival, we investigated the antiapoptosis effect of SR. Compared with the control treatment, Ox-LDL treatment up-regulated the expression of the proapoptotic proteins Bax and Smac and simultaneously down-regulated the expression of the antiapoptotic proteins Bcl2 and cIAP-2. Preincubation with SalB, Re, or SR reversed these effects. SR exhibited the strongest regulatory activity among all three treatments, and Re exhibited stronger regulatory activity than SalB. However, treatment with anisomycin or LY294002 significantly weakened the regulatory ability of SR (**Figures 12A–D**).

We used a flow cytometer to test the activity of cleaved caspase-3 (**Figure 12E**). We found that the activity of cleaved caspase-3 increased under Ox-LDL treatment relative to that under the control treatment. This effect, however, was reversed through incubation with SalB, Re, or SR. The regulatory effects of SalB, Re, and SR on the activity of cleaved caspase-3 are similar to their regulatory effects on the expression of proapoptotic proteins. These results indicated that SR protected HUVECs against Ox-LDL-induced apoptosis through the p38MAPK/NF-κB and PI3K/Akt/NF-κB pathways.

## Discussion

Atherosclerosis is a chronic inflammatory and multifactorial disease. Its treatment through fixed-dose combination therapy has received increased attention in Western medicine (Anderson et al., 2017; Bahiru et al., 2017). For 100s of years, TCM formulations, such as Guanxin Danshen formula, have been used to treat atherosclerosis; these formulations are prepared by combining medicinal herbs in accordance with the rule of *Jun–Chen–Zuo–Shi* (Liu et al., 2015). These formulations exert antiatherogenic effects through the synergistic effects of multiple components on multiple targets. However, understanding and elaborating the mechanism underlying the antiatherogenic effects of TCM formulations remain difficult. Studies on TCM conducted during the past decade have focused on the isolation, purification, structural identification, and pharmacological activity of TCM formulations. However, the simple quantitative analysis of one or several active components of TCM formulations is not in line with the principle of TCM treatment. Thus, a systematic method that could not only validate the efficacy of TCM formulations but also reflect the principle of TCM treatment should be established. In this study, we first used HUVECs to construct an Ox-LDL-induced apoptosis model, which reflects the pathophysiology of atherosclerosis (Orekhov and Ivanova, 2016). Then, we used this model to screen for antiapoptotic components from *S. miltiorrhizae* and *Notoginseng Radix*, the two pharmaceutically active herbs in the Guanxin Danshen formulation. Next, we optimized the combination of the active ingredients of SalB and Re through RSM and used the value of the CI to explore interactions among the identified active components. Furthermore, we analyzed the antiapoptotic mechanism of the compounds through network pharmacology and utilized an *in vitro* model to validate our conclusion.

Guanxin Danshen formula is a TCM preparation used to treat cardiovascular diseases. It is included in the 1995 edition of the Chinese Pharmacopeia. The main therapeutic components of this formula is from *S. miltiorrhizae* and *Notoginseng Radix*; these components can be used to treat atherosclerosis (Sedighi et al., 2017), but it is not clearly what kind of the active substances produce therapeutic effects on anti-atherosclerosis? How make up a prescription can exert most effective protection on anti-atherosclerosis? Our screening results revealed that SalB, a water-soluble component of *S. miltiorrhizae*, and Re, a water-soluble component of *Notoginseng Radix*, exert protective effects against Ox-LDL-induced apoptosis in HUVECs. Consistent with the results of previous studies, our single-factor analysis showed that 12 h of incubation with 59.05–90 μg/mL of SalB and 102.4–160 μg/mL Re provide optimal protective effects against apoptosis (Huang et al., 2016; Chen et al., 2017). However, previous research has focused solely on the simple quantitative analysis of one component to identify the fundamental mechanisms of the pharmaceutical effects of Guanxin Danshen formula. Therefore, we further adopted RSM, which has a rich history in non-linear combinations (Fang et al., 2008; Hather et al., 2013), to systematically study the combination of the active components of SalB and Re. The generated three-dimensional response surfaces and two-dimensional contour plots indicated that SalB and Re mutually interact and exert a quadratic effect on responses (Minto et al., 2000). The generated response surface suggested that SalB and Re in SR synergistically protect HUVECs from Ox-LDL-induced apoptosis. This finding is consistent with the ANOVA of the fitted quadratic model and was validated on the basis of the CI value (Chou, 2006). Furthermore, the antiapoptic effect of SR is stronger than that of SalB or Re alone, as validated in different cell models. Moreover, the synergistic effects of SalB and Re are supported by the combination of *S. miltiorrhiza* and *Notoginseng Radix* (Zhou et al., 2016).

RSM results and the CI value indicated that the effect of SR is more intense than that of SalB or Re alone. Nevertheless, its therapeutic mechanism remained unclear. Therefore, we performed network pharmacology analysis to investigate the molecular mechanisms of action of SR (Hopkins, 2008). We found that SalB and Re have 111 and 69 target candidate genes, respectively, in the endotheliocyte model of Ox-LDL-induced apoptosis. Interestingly, SalB and Re share 37 common targets. Target enrichment analysis and gene–gene interaction networks showed that the main signal pathways of oxidative stress and inflammation are regulated by the shared target genes of SalB and Re. These genes include *GPX1, GPX4, GPX3, SOD1, SOD3, SOD2*, and *MAPKs* and are implicated in the Ox-LDL-induced apoptosis of HUVECs (Li and Mehta, 2009; Lee et al., 2010; Ahsan et al., 2015). Through the analysis of target pathways and disease networks, we found that SalB or Re mediates the same signaling pathways or the same disease network through their specific targets. This finding again proved that SalB and Re in SR synergistically act to protect HUVECs against Ox-LDL-induced apoptosis. The specific target genes of SalB mainly regulate the signal pathways of oxidative stress and inflammation through *PI3K, MAPKs*, and *MMPs*. *PI3K* is the key gene of the PI3K/Akt pathway, and *p38MAPK* is a type of *MAPK*. The PI3K/Akt pathway could regulate apoptosis and antioxidative responses (Lee et al., 2010; Yang et al., 2017). The p38MAPK pathway can adjust the secretion of inflammatory mediators (Li and Mehta, 2009), such as MMPs, ICAM-1, VACM-1, and MCP-1. Moreover, bioinformatics analysis revealed that the specific target genes of Re, such as *AR, ESRs*, and *PPAR*, mainly regulate hormone-related pathways. Re is a phytoestrogen, and evidence indicates that phytoestrogens exert cardiovascular protective and antiapoptotic effects through hormone receptors (Cos et al., 2003; Gencel et al., 2012), such as AR and ESRs (Chakrabarti et al., 2014), which was in good agreement with the network analysis of Re.

Network pharmacology analysis revealed that SR protects endotheliocytes against Ox-LDL-induced apoptosis by regulating the signaling pathways of oxidative stress and inflammation. We proved that SR can markedly down-regulate the expression levels of LOX-1 and NOX4; up-regulate the expression of ERα; reduce the generation of ROS; promote the activities of the antioxidant enzymes SOD, GSH-Px, and CAT; inhibit the expression levels of ICAM-1 and VCAM-1; decrease the expression levels of IL-6, TNF-α, and MCP-1; inhibit the phosphorylation of p38MAPK and the activation of the PI3K/Akt pathway; accelerate the activation of NF-κB; decrease the expression levels of apoptosis proteins and inflammatory mediators; increase the expression levels of antiapoptotic proteins; and finally prevent the Ox-LDL-induced apoptosis of HUVECs. These results are consistent with the network pharmacology analysis and correspond with the pathological changes associated with atherosclerosis that Ox-LDL-induced endotheliocyte apoptosis is the initial factor of atherosclerosis (Asai et al., 2000; Chen et al., 2005; Hansson, 2005), and oxidative stress and inflammation are two critical factors in the Ox-LDL-induced apoptosis of HUVECs (Li and Mehta, 2009; Lee et al., 2010; Ahsan et al., 2015). Pretreatment with anisomycin or LY294002 weakened the protective effect of SR against the Ox-LDL-induced apoptosis of HUVECs. Data indicated that SR protects HUVECs against Ox-LDL-induced apoptosis through the PI3K/Akt and p-p38MAPK/p38MAPK pathways. Nevertheless, this study also has disadvantages. For example, the effect of SalB on the expression of LOX-1 and ERα should be further investigated. Similarly, the effect of Re on the expression of LOX-1 and NOX4 should be tested further, and the protective effect of SR should be validated *in vivo*.

## Conclusion

**FIGURE 13:**
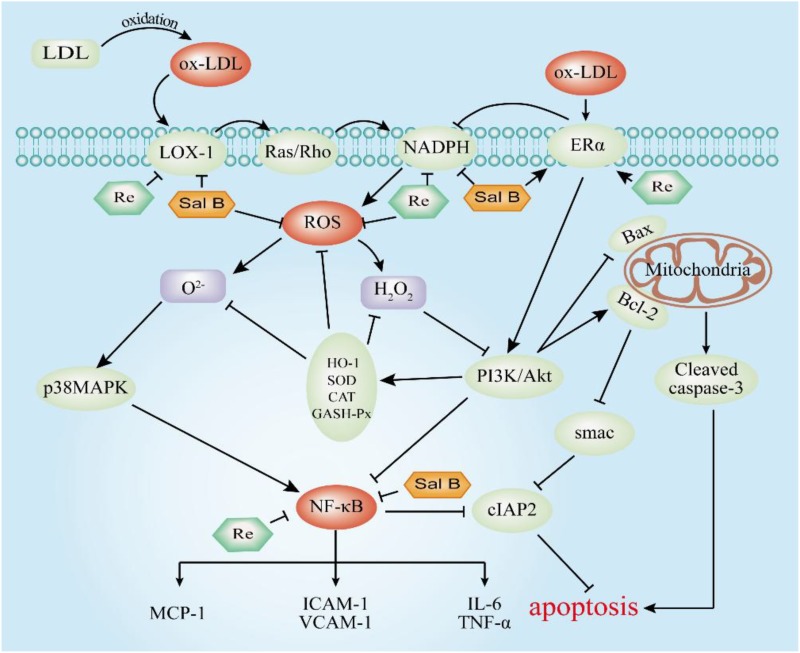
Molecular mechanism underlying the antiapoptotic effect of SR. T-bars represent inhibition, and arrows represent activation.

To summarize (**Figure 13**), we demonstrated that SalB and Re in SR could synergistically protect HUVECs against Ox-LDL-induced apoptosis. SalB and Re in SR exert their synergistic antiapoptotic effects by up-regulating the activities of antioxidant enzymes, inhibiting the secretion of inflammatory factors, and restoring the balanced expression of proapoptotic and antiapoptotic proteins through the p38MAPK/NF-κB and PI3K/Akt/NF-κB pathways, which are mediated by the receptors of LOX-1, NOX4, and ERα. Our findings indicated that SR may be an effective treatment against endothelial cell apoptosis.

## Author Contributions

KY performed the major experiments and wrote this paper. YL checked the results of the experiments. SL revised the manuscript. RH was responsible for network pharmacology. YD and PL were in charge of screening active compounds. GS and XS answered for designing experiments.

## Conflict of Interest Statement

The authors declare that the research was conducted in the absence of any commercial or financial relationships that could be construed as a potential conflict of interest.

## Footnotes

1 http://www.genecards.org/

2 http://idtarget.rcas.sinica.edu.tw/

3 http://lilab.ecust.edu.cn/pharmmapper/

4 https://david.ncifcrf.gov/

5 https://www.biostars.org/p/200126/

6 https://string-db.org/

## References

[B1] Ahsan A. Han G. Pan J. Liu S. Padhiar A. A. Chu P. (2015). Phosphocreatine protects endothelial cells from oxidized low-density lipoprotein-induced apoptosis by modulating the PI3K/Akt/eNOS pathway. *Apoptosis* 20 1563–1576. 10.1007/s10495-015-1175-4 26404526

[B2] Anderson D. Liu R. Anand Subramony J. Cammack J. (2017). Design control considerations for biologic-device combination products. *Adv. Drug Deliv. Rev.* 112 101–105. 10.1016/j.addr.2017.01.003 28088344

[B3] Asai K. Kudej R. K. Shen Y. T. Yang G. P. Takagi G. Kudej A. B. (2000). Peripheral vascular endothelial dysfunction and apoptosis in old monkeys. *Arterioscler. Thromb. Vasc. Biol.* 20 1493–1499. 10.1161/01.ATV.20.6.1493 10845863

[B4] Bahiru E. De Cates A. N. Farr M. R. Jarvis M. C. Palla M. Rees K. (2017). Fixed-dose combination therapy for the prevention of atherosclerotic cardiovascular diseases. *Cochrane Database Syst. Rev.* 3:CD009868. 10.1002/14651858.CD009868.pub3 28263370 PMC6464321

[B5] Chakrabarti S. Morton J. S. Davidge S. T. (2014). Mechanisms of estrogen effects on the endothelium: an overview. *Can. J. Cardiol.* 30 705–712. 10.1016/j.cjca.2013.08.006 24252499

[B6] Chen F. Eriksson P. Kimura T. Herzfeld I. Valen G. (2005). Apoptosis and angiogenesis are induced in the unstable coronary atherosclerotic plaque. *Coron. Artery Dis.* 16 191–197. 10.1097/00019501-200505000-00009 15818089

[B7] Chen H. M. Luo H. Zeng W. B. Liu B. Huang J. C. Liu M. (2017). Salvianolic acid B attenuates oxidized low-density lipoprotein-induced endothelial cell apoptosis through inhibition of oxidative stress, p53, and caspase-3 pathways. *Chin. J. Integr. Med.* 10.1007/s11655-016-2645-4 [Epub ahead of print]. 28116660

[B8] Chen J. Mehta J. L. Haider N. Zhang X. Narula J. Li D. (2004). Role of caspases in Ox-LDL-induced apoptotic cascade in human coronary artery endothelial cells. *Circ. Res.* 94 370–376. 10.1161/01.RES.0000113782.07824.BE 14684629

[B9] Chen X. P. Xun K. L. Wu Q. Zhang T. T. Shi J. S. Du G. H. (2007). Oxidized low density lipoprotein receptor-1 mediates oxidized low density lipoprotein-induced apoptosis in human umbilical vein endothelial cells: role of reactive oxygen species. *Vascul. Pharmacol.* 47 1–9. 10.1016/j.vph.2007.01.004 17433786

[B10] Chou T. C. (2006). Theoretical basis, experimental design, and computerized simulation of synergism and antagonism in drug combination studies. *Pharmacol. Rev.* 58 621–681. 10.1124/pr.58.3.10 16968952

[B11] Cos P. De Bruyne T. Apers S. Vanden Berghe D. Pieters L. Vlietinck A. J. (2003). Phytoestrogens: recent developments. *Planta Med.* 69 589–599. 10.1055/s-2003-41122 12898412

[B12] Fang H. B. Ross D. D. Sausville E. Tan M. (2008). Experimental design and interaction analysis of combination studies of drugs with log-linear dose responses. *Stat. Med.* 27 3071–3083. 10.1002/sim.3204 18186545

[B13] Gencel V. B. Benjamin M. M. Bahou S. N. Khalil R. A. (2012). Vascular effects of phytoestrogens and alternative menopausal hormone therapy in cardiovascular disease. *Mini Rev. Med. Chem.* 12 149–174. 10.2174/138955712798995020 22070687 PMC3288319

[B14] Han J. Y. Fan J. Y. Horie Y. Miura S. Cui D. H. Ishii H. (2008). Ameliorating effects of compounds derived from *Salvia miltiorrhiza* root extract on microcirculatory disturbance and target organ injury by ischemia and reperfusion. *Pharmacol. Ther.* 117 280–295. 10.1016/j.pharmthera.2007.09.008 18048101

[B15] Hansson G. K. (2005). Inflammation, atherosclerosis, and coronary artery disease. *N. Engl. J. Med.* 352 1685–1695. 10.1056/NEJMra043430 15843671

[B16] Hather G. Chen H. H. Liu R. (2013). Experimental design for in vitro drug combination studies. *Top. Appl. Stat.* 55 337–344. 10.1007/978-1-4614-7846-1_27

[B17] Hopkins A. L. (2008). Network pharmacology: the next paradigm in drug discovery. *Nat. Chem. Biol.* 4 682–690. 10.1038/nchembio.118 18936753

[B18] Huang G. D. Zhong X. F. Deng Z. Y. Zeng R. (2016). Proteomic analysis of ginsenoside Re attenuates hydrogen peroxide-induced oxidative stress in human umbilical vein endothelial cells. *Food Funct.* 7 2451–2461. 10.1039/c6fo00123h 27161858

[B19] Isgut M. Rao M. Yang C. Subrahmanyam V. Rida P. C. G. Aneja R. (2017). Application of combination high-throughput phenotypic screening and target identification methods for the discovery of natural product-based combination drugs. *Med. Res. Rev.* 38 504–524. 10.1002/med.21444 28510271

[B20] Lee H. J. Seo M. Lee E. J. (2014). Salvianolic acid B inhibits atherogenesis of vascular cells through induction of Nrf2-dependent heme oxygenase-1. *Curr. Med. Chem.* 21 3095–3106. 10.2174/0929867321666140601195940 24934350

[B21] Lee W. J. Ou H. C. Hsu W. C. Chou M. M. Tseng J. J. Hsu S. L. (2010). Ellagic acid inhibits oxidized LDL-mediated LOX-1 expression, ROS generation, and inflammation in human endothelial cells. *J. Vasc. Surg.* 52 1290–1300. 10.1016/j.jvs.2010.04.085 20692795

[B22] Li D. Mehta J. L. (2009). Intracellular signaling of LOX-1 in endothelial cell apoptosis. *Circ. Res.* 104 566–568. 10.1161/CIRCRESAHA.109.194209 19286611

[B23] Li M. Zhao M. Q. Kumar Durairajan S. S. Xie L. X. Zhang H. X. Kum W. F. (2008). Protective effect of tetramethylpyrazine and salvianolic acid B on apoptosis of rat cerebral microvascular endothelial cell under high shear stress. *Clin. Hemorheol. Microcirc.* 38 177–187. 18239260

[B24] Li P. Lv B. Jiang X. Wang T. Ma X. Chang N. (2016). Identification of NF-kappaB inhibitors following Shenfu injection and bioactivity-integrated UPLC/Q-TOF-MS and screening for related anti-inflammatory targets in vitro and in silico. *J. Ethnopharmacol.* 194 658–667. 10.1016/j.jep.2016.10.052 27771457

[B25] Li S. L. Song J. Z. Qiao C. F. Zhou Y. Qian K. Lee K. H. (2010). A novel strategy to rapidly explore potential chemical markers for the discrimination between raw and processed Radix Rehmanniae by UHPLC-TOFMS with multivariate statistical analysis. *J. Pharm. Biomed. Anal.* 51 812–823. 10.1016/j.jpba.2009.10.002 19879709

[B26] Liao P. Sun G. Zhang C. Wang M. Sun Y. Zhou Y. (2016). Bauhinia championii flavone attenuates hypoxia-reoxygenation induced apoptosis in H9c2 cardiomyocytes by improving mitochondrial dysfunction. *Molecules* 21:E1469. 10.3390/molecules21111469 27827932 PMC6273835

[B27] Liu Q. Li J. Hartstone-Rose A. Wang J. Li J. Janicki J. S. (2015). Chinese herbal compounds for the prevention and treatment of atherosclerosis: experimental evidence and mechanisms. *Evid. Based Complement. Altern. Med.* 2015:752610. 10.1155/2015/752610 26089946 PMC4451781

[B28] Martinet W. Kockx M. M. (2001). Apoptosis in atherosclerosis: focus on oxidized lipids and inflammation. *Curr. Opin. Lipidol.* 12 535–541. 10.1097/00041433-200110000-00009 11561173

[B29] Minto C. F. Schnider T. W. Short T. G. Gregg K. M. Gentilini A. Shafer S. L. (2000). Response surface model for anesthetic drug interactions. *Anesthesiology* 92 1603–1616. 10.1097/00000542-200006000-00017 10839909

[B30] Orekhov A. N. Ivanova E. A. (2016). Cellular models of atherosclerosis and their implication for testing natural substances with anti-atherosclerotic potential. *Phytomedicine* 23 1190–1197. 10.1016/j.phymed.2016.01.003 26922038

[B31] Peng L. Sun S. Xie L. H. Wicks S. M. Xie J. T. (2012). Ginsenoside Re: pharmacological effects on cardiovascular system. *Cardiovasc. Ther.* 30 e183–e188. 10.1111/j.1755-5922.2011.00271.x 21884006

[B32] Peters S. A. Den Ruijter H. M. Bots M. L. Moons K. G. (2012). Improvements in risk stratification for the occurrence of cardiovascular disease by imaging subclinical atherosclerosis: a systematic review. *Heart* 98 177–184. 10.1136/heartjnl-2011-300747 22095617

[B33] Qin M. Luo Y. Meng X. B. Wang M. Wang H. W. Song S. Y. (2015). Myricitrin attenuates endothelial cell apoptosis to prevent atherosclerosis: an insight into PI3K/Akt activation and STAT3 signaling pathways. *Vascul. Pharmacol.* 70 23–34. 10.1016/j.vph.2015.03.002 25849952

[B34] Ren Y. Tao S. Zheng S. Zhao M. Zhu Y. Yang J. (2016). Salvianolic acid B improves vascular endothelial function in diabetic rats with blood glucose fluctuations via suppression of endothelial cell apoptosis. *Eur. J. Pharmacol.* 791 308–315. 10.1016/j.ejphar.2016.09.014 27614127

[B35] Ross R. (1999). Atherosclerosis–an inflammatory disease. *N. Engl. J. Med.* 340 115–126. 10.1056/NEJM199901143400207 9887164

[B36] Sedighi M. Bahmani M. Asgary S. Beyranvand F. Rafieian-Kopaei M. (2017). A review of plant-based compounds and medicinal plants effective on atherosclerosis. *J. Res. Med. Sci.* 22:30. 10.4103/1735-1995.202151 28461816 PMC5390544

[B37] Sun B. Li C. Zuo L. Liu P. (2016). Protection of SAL B with H9C2 cells. *Pharm. Biol.* 54 889–895. 10.3109/13880209.2015.1089911 26705025

[B38] Sun G. B. Qin M. Ye J. X. Pan R. L. Meng X. B. Wang M. (2013). Inhibitory effects of myricitrin on oxidative stress-induced endothelial damage and early atherosclerosis in ApoE-/- mice. *Toxicol. Appl. Pharmacol.* 271 114–126. 10.1016/j.taap.2013.04.015 23639522

[B39] Sun X. Sun G. B. Wang M. Xiao J. Sun X. B. (2011). Protective effects of cynaroside against H_2_O_2_-induced apoptosis in H9c2 cardiomyoblasts. *J. Cell. Biochem.* 112 2019–2029. 10.1002/jcb.23121 21445859

[B40] Yang K. Zhang H. Luo Y. Zhang J. Wang M. Liao P. (2017). Gypenoside XVII prevents atherosclerosis by attenuating endothelial apoptosis and oxidative stress: insight into the ERalpha-Mediated PI3K/Akt pathway. *Int. J. Mol. Sci.* 18:77. 10.3390/ijms18020077 28208754 PMC5343768

[B41] Zhou X. Razmovski-Naumovski V. Chang D. Li C. Kam A. Low M. (2016). Synergistic effects of Danshen (Salvia Miltiorrhiza Radix et Rhizoma) and Sanqi (Notoginseng Radix et Rhizoma) combination in inhibiting inflammation mediators in RAW264.7 Cells. *Biomed Res. Int.* 2016:5758195. 10.1155/2016/5758195 27830149 PMC5088307

