# Supplementary material for: Salvianolic Acid B and Ginsenoside Re Synergistically Protect Against Ox-LDL-Induced Endothelial Apoptosis Through the Antioxidative and Antiinflammatory Mechanisms
Source: Front Pharmacol. 2018 Jun 20;9:662. doi: 10.3389/fphar.2018.00662 (PMC6019702; doi:10.3389/fphar.2018.00662)
Supplement: Supplementary file 1 [file Presentation_1.ZIP › supplemental material/supplemental material 1.PDF]

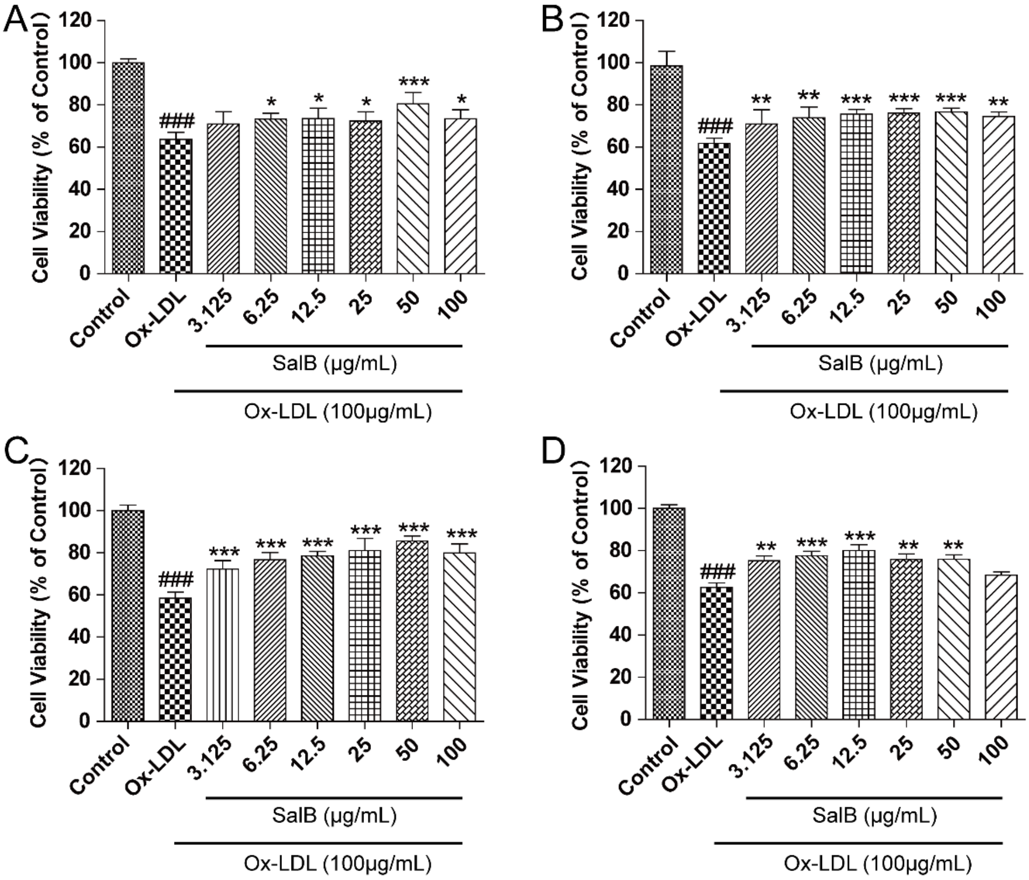

Figure 1: Cytoprotective effects for SalB on Ox-LDL-induced cytotoxicity in HUVECs. (a) Incubation with SalB for 4 h significantly lowered Ox-LDL-induced cell injury. Cell viability was measured by MTT assay. (b) Incubation with SalB for 8 h significantly lowered Ox-LDL-induced cell injury. Cell viability was measured by MTT assay. (c) Incubation with SalB for 12 h significantly lowered Ox-LDL-induced cell injury. Cell viability was measured by MTT assay. (d) Incubation with SalB for 24 h significantly lowered Ox-LDL-induced cell injury. Cell viability was measured by MTT assay. The values are expressed as the mean ± SD. from three independent experiments. ###*P < 0.001 vs Control; *P < 0.05, **P < 0.01, ***P < 0.001 vs Model*.

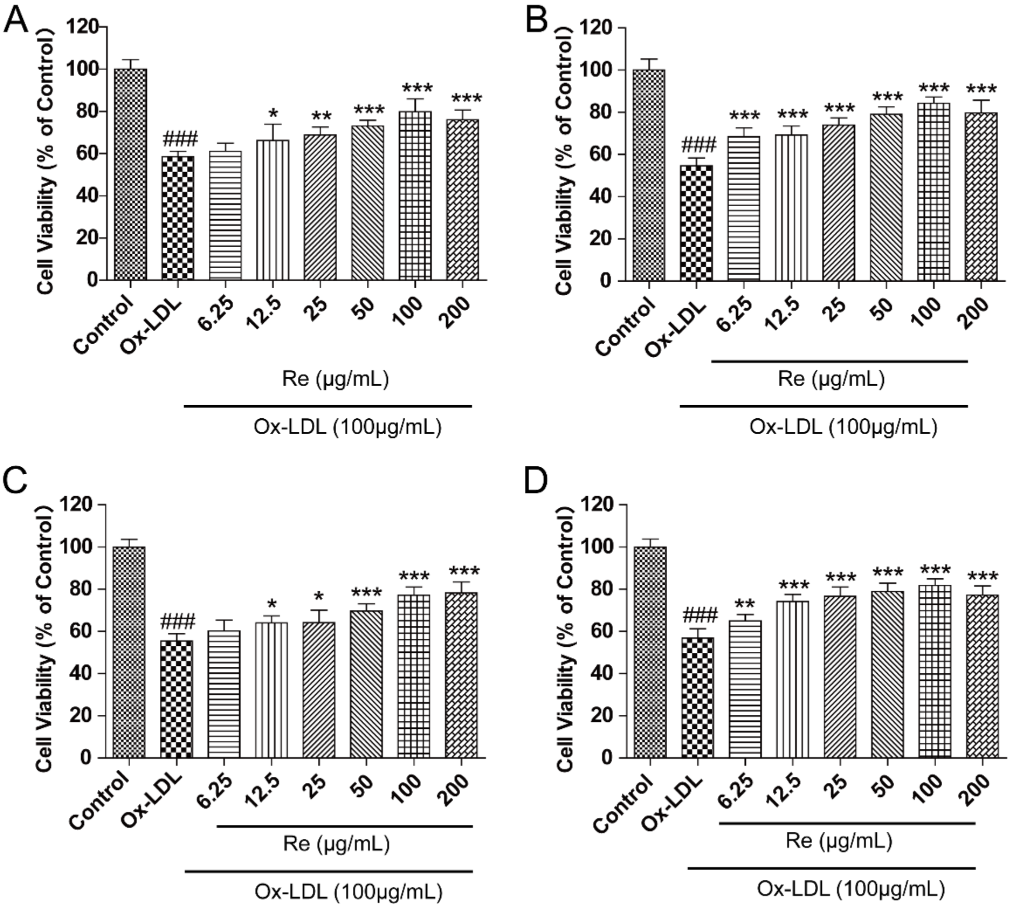

Figure 2: Cytoprotective effects for Re on Ox-LDL-induced cytotoxicity in HUVECs. (a) Incubation with Re for 4 h significantly lowered Ox-LDL-induced cell injury. Cell viability was measured by MTT assay. (b) Incubation with Re for 8 h significantly lowered Ox-LDL-induced cell injury. Cell viability was measured by MTT assay. (c) Incubation with Re for 12 h significantly lowered Ox-LDL-induced cell injury. Cell viability was measured by MTT assay. (d) Incubation with Re for 24 h significantly lowered Ox-LDL-induced cell injury. Cell viability was measured by MTT assay. The values are expressed as the mean ± SD. from three independent experiments. $^{\#\#\#}P < 0.001$ *vs Control*; $*P < 0.05$, $**P < 0.01$, $***P < 0.001$ *vs Model*.

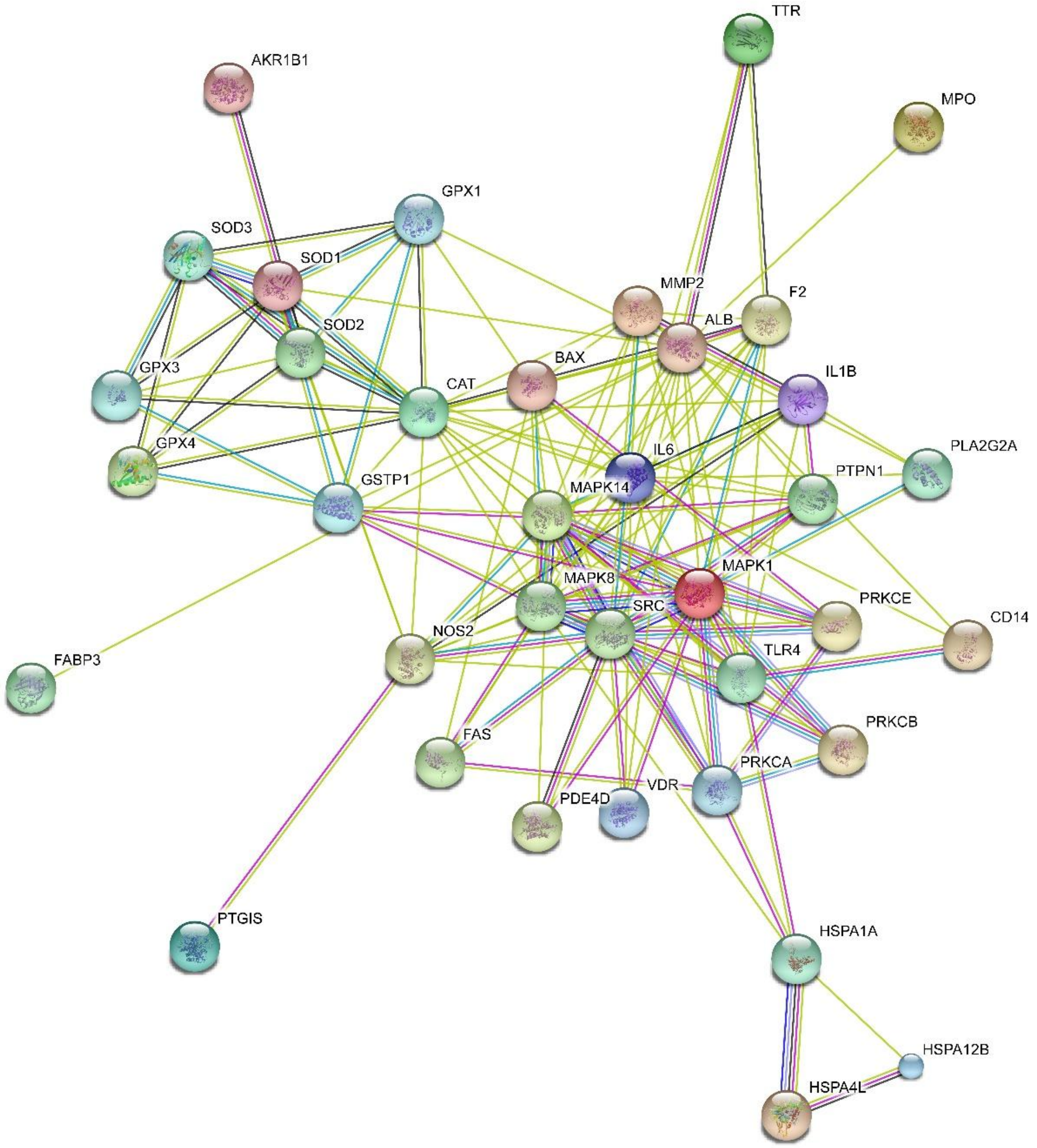

Figure 3: Interaction of the target genes shared by salvianolic acid B and ginsenoside Re.

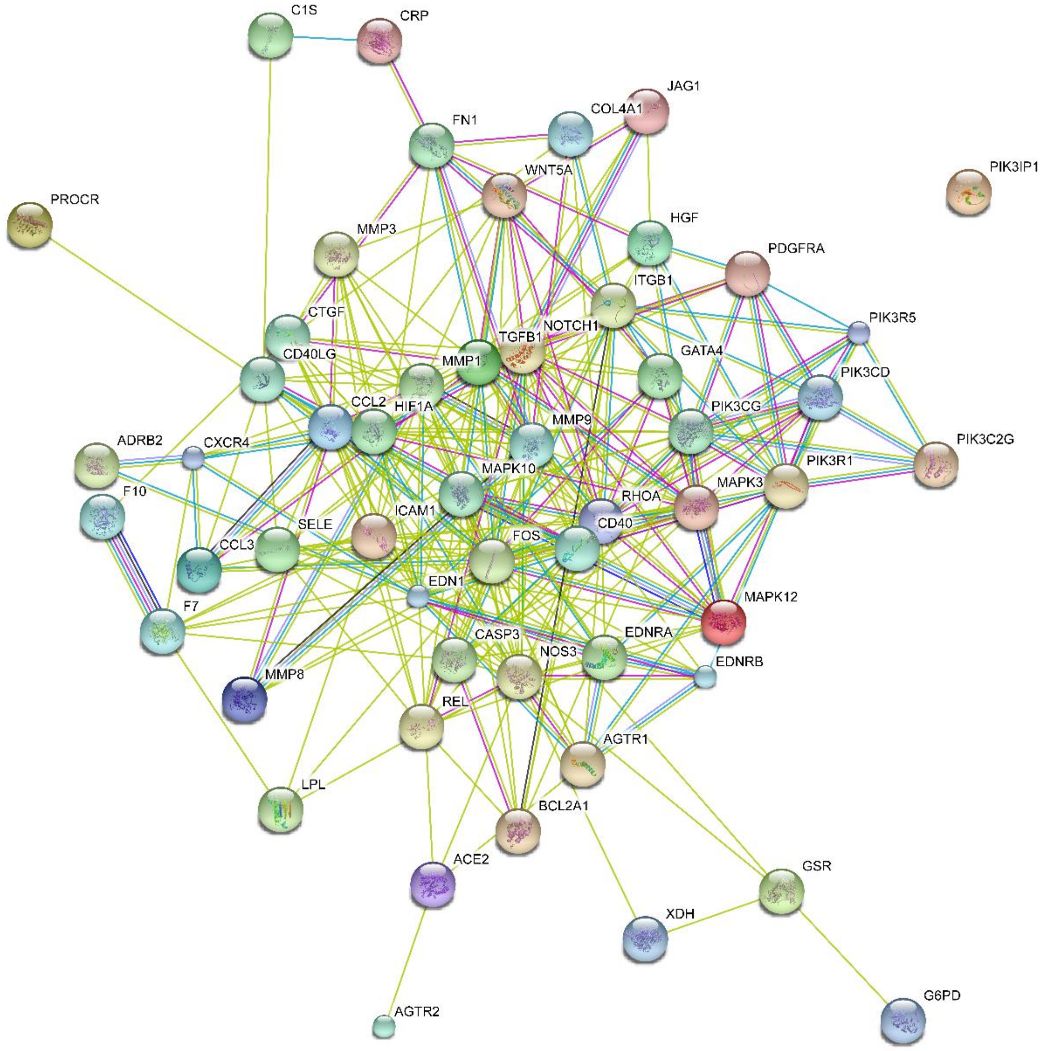

Figure 4: Interaction of specific target genes of salvianolic acid B.

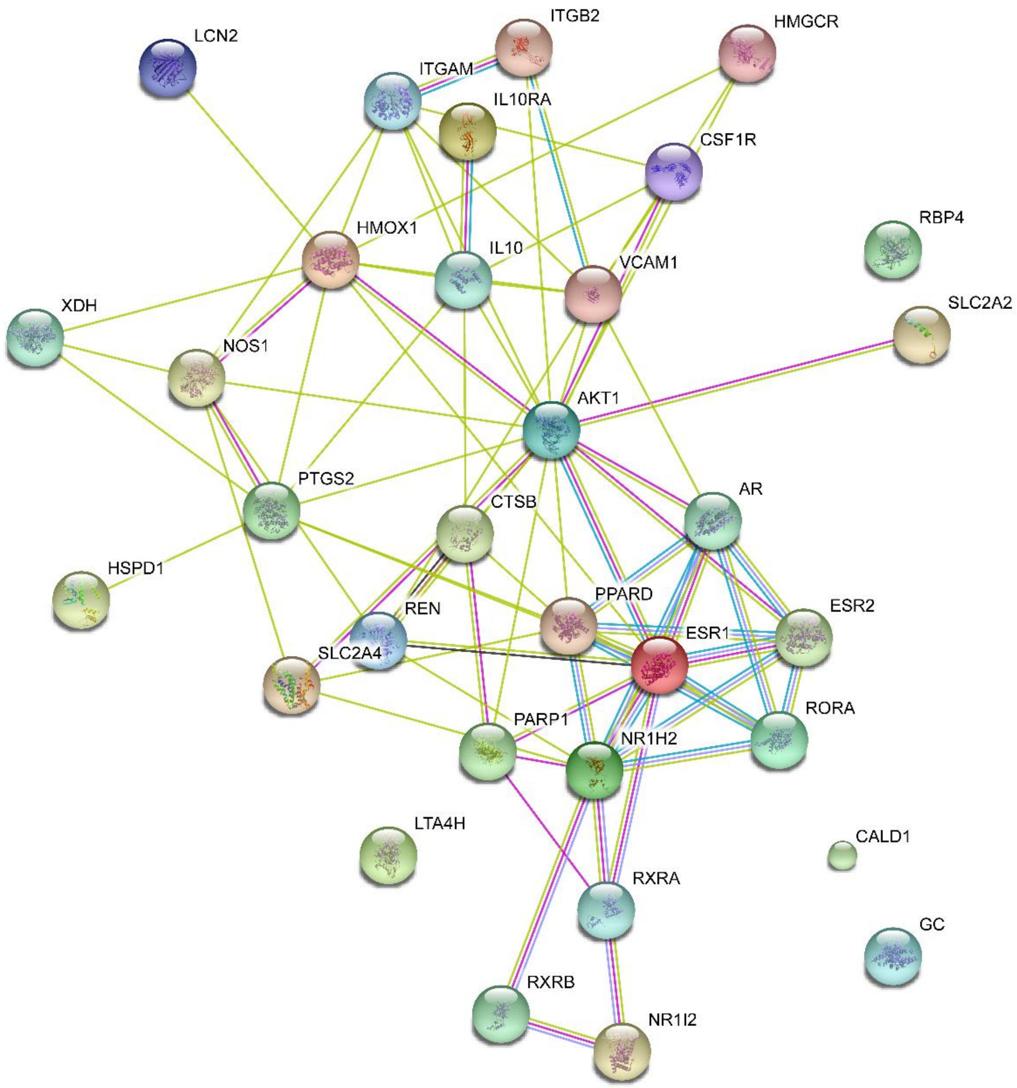

Figure 5: Interaction of specific target genes of ginsenoside Re.
