# Supplementary material for: Salvianolic Acid B and Ginsenoside Re Synergistically Protect Against Ox-LDL-Induced Endothelial Apoptosis Through the Antioxidative and Antiinflammatory Mechanisms
Source: Front Pharmacol. 2018 Jun 20;9:662. doi: 10.3389/fphar.2018.00662 (PMC6019702; doi:10.3389/fphar.2018.00662)
Supplement: Supplementary file 1 [file Presentation_1.ZIP › supplemental material/supplemental material 2.PDF]

the 2^k factor design experiment

The two factors investigated were: SalB; Re. The influence of variations in these factors on the cell viability was evaluated using a 2^k factorial design (Table.1). A total of 4 experimental runs were conducted (randomly) for which 3 replicates were performed. The outputs of the experimental design were analysed using Design Expert 8.0.6 software (Stat-Ease, Inc.). The magnitude and direction of factor effect were determined via OD calculations and the significance of the factor effect was evaluated using ANOVA (Table.2).

Table.1 2^k factorial experiment: Salvianolic acid B and ginsenoside Re dosage

SalB (μg/mL)	Re (μg/mL)	OD
50	140	0.68112
100	70	0.59444
50	70	0.63527
100	140	0.65002

Result

The regression analysis with Design Expert 8.0.6 software (Stat-Ease, Inc.) fits the 2^k factorial design data into the following equations:

Final Equation in Terms of Coded Factors: $R = 0.62 - 0.022 * A + 0.027 * B$

Final Equation in Terms of Actual Factors: $R = 0.60905 - 8.92950E - 004 * A + 7.66250E - 004 * B$

R represented the value of OD, A represented SalB, B represented Re.

Table.2 Significance test of regression coefficients for 2^k factorial experimental equation

Source	Sum of Squares	df	Mean Square	*F Value*	*p-value Prob > F*	significant
Model	0.019	2	9.741E-003	11.28	0.0014	**
A	7.974E-003	1	7.974E-003	9.23	0.0095	**
B	0.012	1	0.012	13.32	0.0029	**
Residual	0.011	13	8.636E-004			
Lack of Fit	1.991E-004	1	1.991E-004	0.22	0.6499	
Pure Error	0.011	12	9.190E-004			
Cor Total	0.031	15				
