# Supplementary material for: Salvianolic Acid B and Ginsenoside Re Synergistically Protect Against Ox-LDL-Induced Endothelial Apoptosis Through the Antioxidative and Antiinflammatory Mechanisms
Source: Front Pharmacol. 2018 Jun 20;9:662. doi: 10.3389/fphar.2018.00662 (PMC6019702; doi:10.3389/fphar.2018.00662)
Supplement: Supplementary file 1 [file Presentation_1.ZIP › supplemental material/supplemental material 3.PDF]

steepest ascent test

Using the first-order mathematical linear equation predicted from the 2^k factorial design, which described the relationship between the responses and factors, step-wise factor concentrations were determined to test along the direction of steepest ascent, which formed a new set of experimental runs. Moving along the path of steepest ascent to a more optimum region for sensitivity involved taking steps that were proportional in size to the parameter b_i values in the fitted first order model.

The path began at the center point of the 2^k factorial design, serving as the origin for the steepest ascent experiment, and stretched outside the original design space to explore the outside region. A sequence of equally spaced steps along the path were selected which formed a set of experimental runs consisting of different coating and detector antibody concentrations. Factor concentrations were either increased or decreased each step as directed by the sign of these terms in the linear model equation. The magnitude of the increase and decrease in concentration was determined by the parameter estimates, SalB and Re in the linear model equation (Supplementary material 2).

SalB and Re concentrations were varied in the experiment outlined in Table 1, with each set of conditions representing an experimental run. Eight runs were evaluated over one plates, with each run replicated three. The cell viability was evaluated. Experimental designs, statistical analysis and model prediction were performed using Design Expert 8.0.6 software (Stat-Ease, Inc.).

Table.1 Climbing test: Salvianolic acid B and ginsenoside Re dosage

Run	scheme	SalB (μg/mL)	Re (μg/mL)	OD
1	zero point (0)	75	105	0.704735
2	step (△)	-4	5	
3	0+1 △	71	110	0.788305
4	0+2 △	67	115	0.81443
**5**	**0+3 △**	**63**	**120**	**0.8356225**
6	0+4 △	59	125	0.7690275
7	0+5 △	55	130	0.7972225
8	0+6 △	51	135	0.7771925

Result

According to the Table.1, the ideal dosage of SR was 63 μg/mL - 120 μg/mL, so they were further used to central composite design.
