# Supplementary material for: Salvianolic Acid B and Ginsenoside Re Synergistically Protect Against Ox-LDL-Induced Endothelial Apoptosis Through the Antioxidative and Antiinflammatory Mechanisms
Source: Front Pharmacol. 2018 Jun 20;9:662. doi: 10.3389/fphar.2018.00662 (PMC6019702; doi:10.3389/fphar.2018.00662)
Supplement: Supplementary file 1 [file Presentation_1.ZIP › supplemental material/supplemental material 4.PDF]

Response surface methodology

Response surface methodology was used to study the effects of independent variables: SalB; (A) and Re (B) on the cell viability (OD). Experimental designs along with coded and un-coded values are given in Table 1. Central Composite Design (CCD) was used along with quadratic model. Each independent variable contains five levels: -1.68, -1, 0, 1 and 1.68. Thirteen combinations, including six replicates at central point was chosen randomly according to central composite designs which are listed in Table 1.

The analysis of variance (ANOVA) carried out to determine quadratic and interaction regression coefficient using Design Expert 8.0.6 software (Stat-Ease, Inc.) and the fitness of the polynomial equation to the responses were estimated using coefficient of determination (R^2). The significance of all the terms of polynomial equation was analyzed statistically by computing the F value at $p < 0.05$ (Table 2). The trial version of Design Expert 8.0.6 software (Stat-Ease, Inc.) was used to generate 3D response surface graphs.

Table.1 Optimization of Compatibility of salvianolic acid B and ginsenoside Re by response surface methodology

Run	SalB (μg/mL)	Re (μg/mL)	OD
1	46	100	0.654798
2	80	100	0.674065
3	46	140	0.792487
4	80	140	0.671063
5	39	120	0.71337
6	87	120	0.665192
7	63	92	0.657268
8	63	148	0.680782
9	63	120	0.80618
10	63	120	0.79084
11	63	120	0.78349
12	63	120	0.80414
13	63	120	0.8143

Result

The regression analysis with Design Expert 8.0.6 software (Stat-Ease, Inc.) fits the steepest ascent test data into the following equations:

Final Equation in Terms of Coded Factors: $R = 0.8 - 0.021 * A + 0.021 * B - 0.035 *$

$A * B - 0.051 * A^2 - 0.061 * B^2$

Final Equation in Terms of Actual Factors: $R = -2.90635 + 0.033186 * A + 0.043954 * B - 1.03449E - 004 * A * B - 1.74799E - 004 * A^2 - 1.51612E - 004 * B^2$

R represented the value of OD, A represented SalB, B represented Re.

Table.2 Analysis of variance in response surface experiment

Source	Sum of Squares	df	Mean Square	*F Value*	*p-value Prob > F*	significant
Model	0.051	5	0.010	25.58	0.0002	***
A	3.625E-003	1	3.625E-003	9.18	0.0191	*
B	3.526E-003	1	3.526E-003	8.93	0.0203	*
AB	4.948E-003	1	4.948E-003	12.53	0.0095	**
A^2	0.018	1	0.018	44.94	0.0003	***
B^2	0.026	1	0.026	64.77	< 0.0001	***
Residual	2.765E-003	7	3.950E-004			
Lack of Fit	2.149E-003	3	7.163E-004	4.65	0.0859	
Pure Error	6.161E-004	4	1.540E-004			
Cor Total	0.053	12				
