# Supplementary material for: Salvianolic Acid B and Ginsenoside Re Synergistically Protect Against Ox-LDL-Induced Endothelial Apoptosis Through the Antioxidative and Antiinflammatory Mechanisms
Source: Front Pharmacol. 2018 Jun 20;9:662. doi: 10.3389/fphar.2018.00662 (PMC6019702; doi:10.3389/fphar.2018.00662)
Supplement: Supplementary file 1 [file Presentation_1.ZIP › supplemental material/supplemental material 6/original image of apoptosis by flow cytometry in paper.pdf]

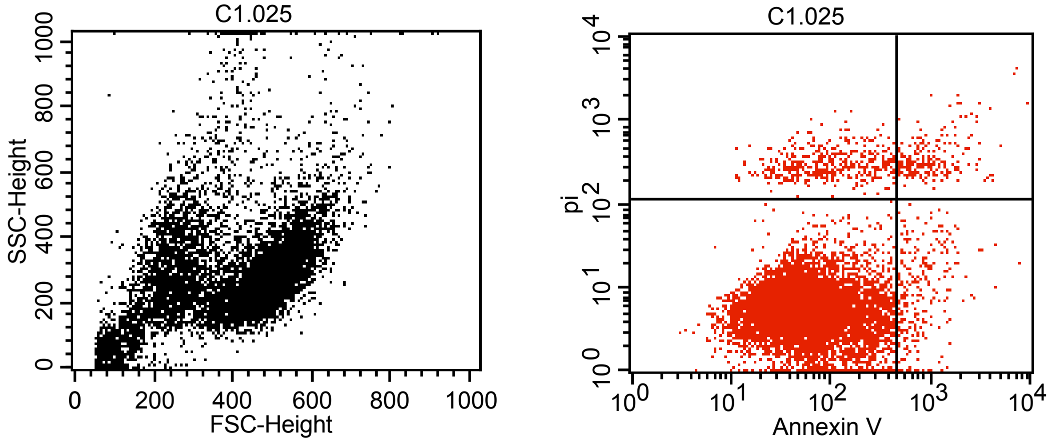

File: C1.025

Quad	Events	% Gated	% Total
UL	441	5.36	4.86
UR	235	2.86	2.59
LL	7259	88.24	79.98
LR	291	3.54	3.21

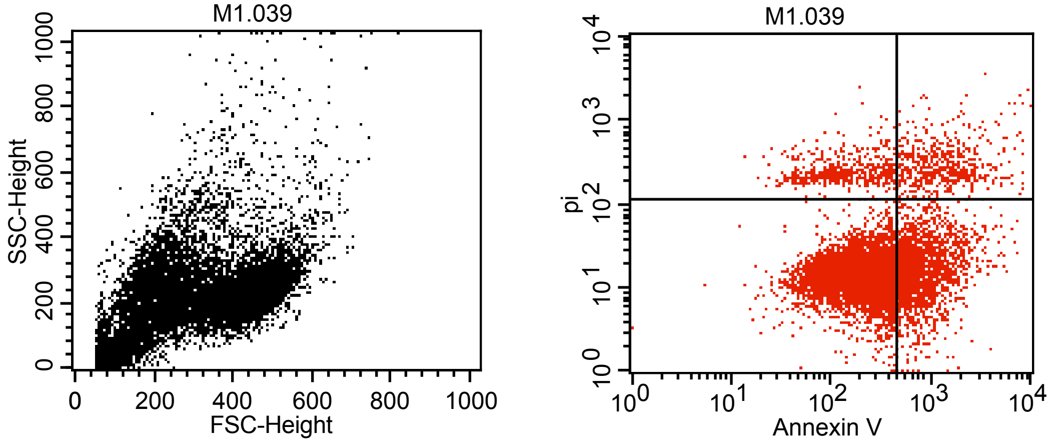

File: M1.039

Quad	Events	% Gated	% Total
UL	534	6.24	5.43
UR	530	6.20	5.38
LL	5540	64.77	56.28
LR	1949	22.79	19.80

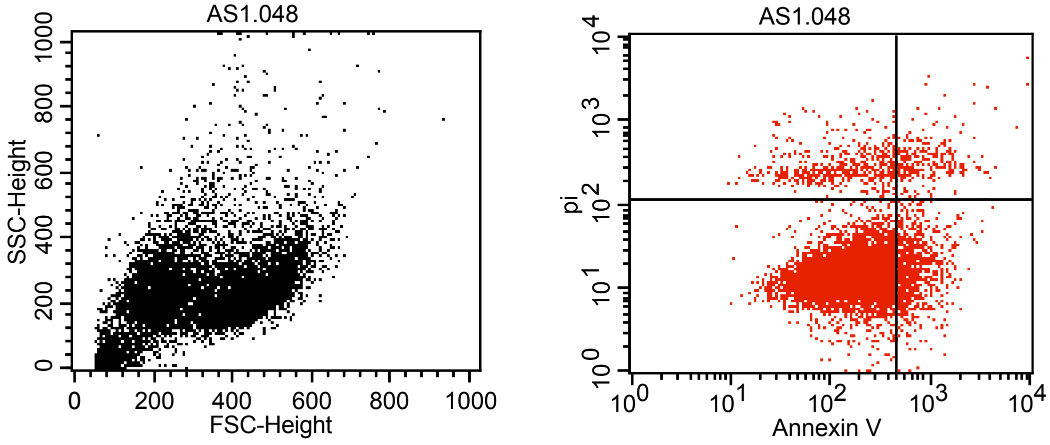

File: AS1.048

Quad	Events	% Gated	% Total
UL	523	6.21	5.60
UR	275	3.27	2.94
LL	6726	79.92	71.97
LR	892	10.60	9.54

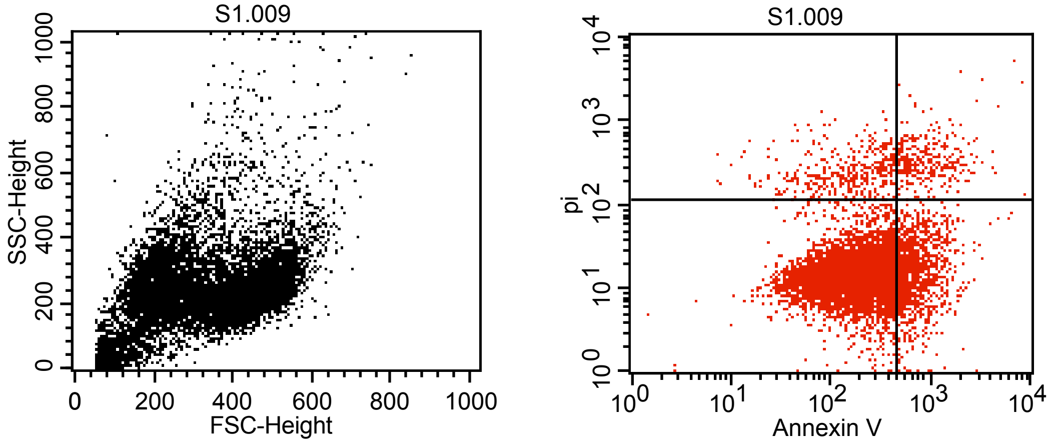

File: S1.009

Quad	Events	% Gated	% Total
UL	391	4.66	4.10
UR	328	3.91	3.44
LL	6435	76.70	67.41
LR	1236	14.73	12.95

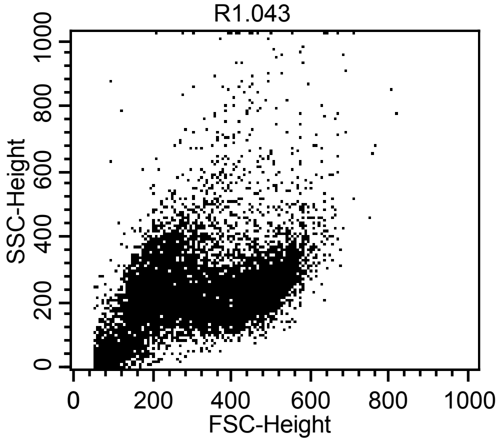

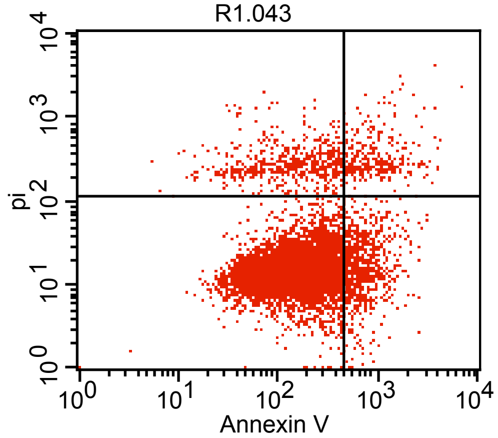

File: R1.043

Quad	Events	% Gated	% Total
UL	547	6.49	5.76
UR	302	3.58	3.18
LL	6774	80.33	71.36
LR	810	9.61	8.53

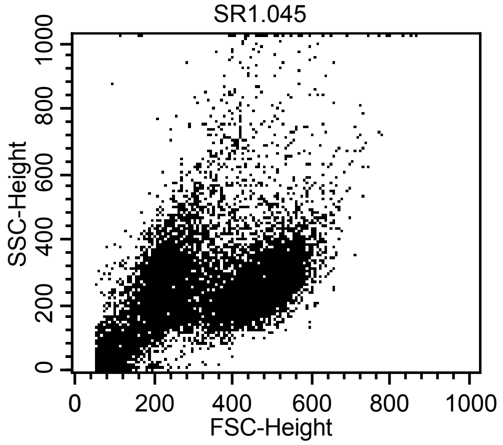

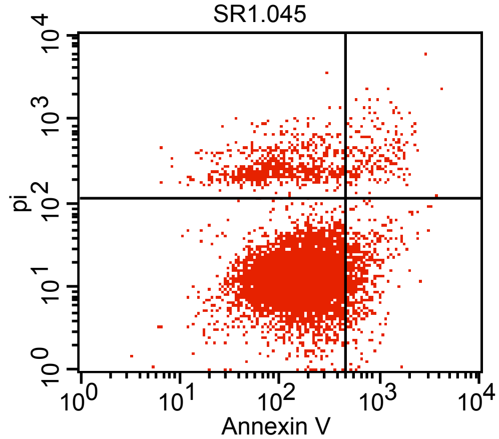

File: SR1.045

Quad	Events	% Gated	% Total
UL	722	8.60	7.38
UR	196	2.33	2.00
LL	6976	83.06	71.34
LR	505	5.15	5.16
