# Supplementary figures and images for: Salvianolic Acid B and Ginsenoside Re Synergistically Protect Against Ox-LDL-Induced Endothelial Apoptosis Through the Antioxidative and Antiinflammatory Mechanisms
Source: Front Pharmacol. 2018 Jun 20;9:662. doi: 10.3389/fphar.2018.00662 (PMC6019702; doi:10.3389/fphar.2018.00662)

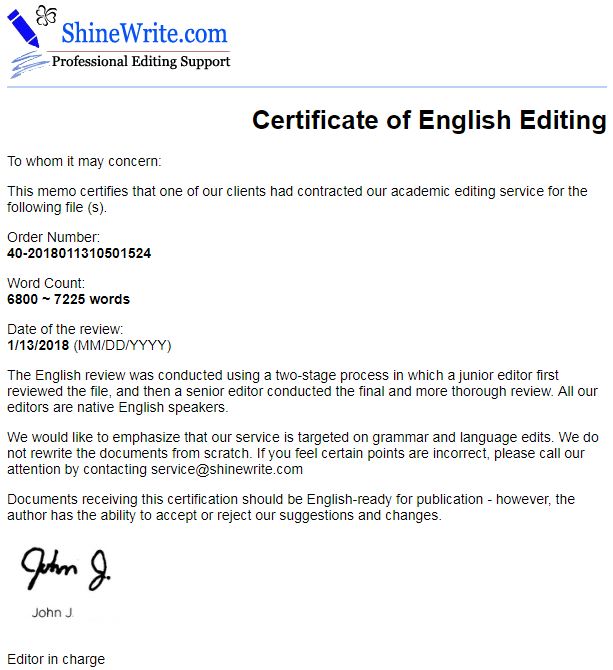

Supplement: Supplementary file 1 [file Presentation_1.ZIP › supplemental material/Certificate of English Editing.JPG]

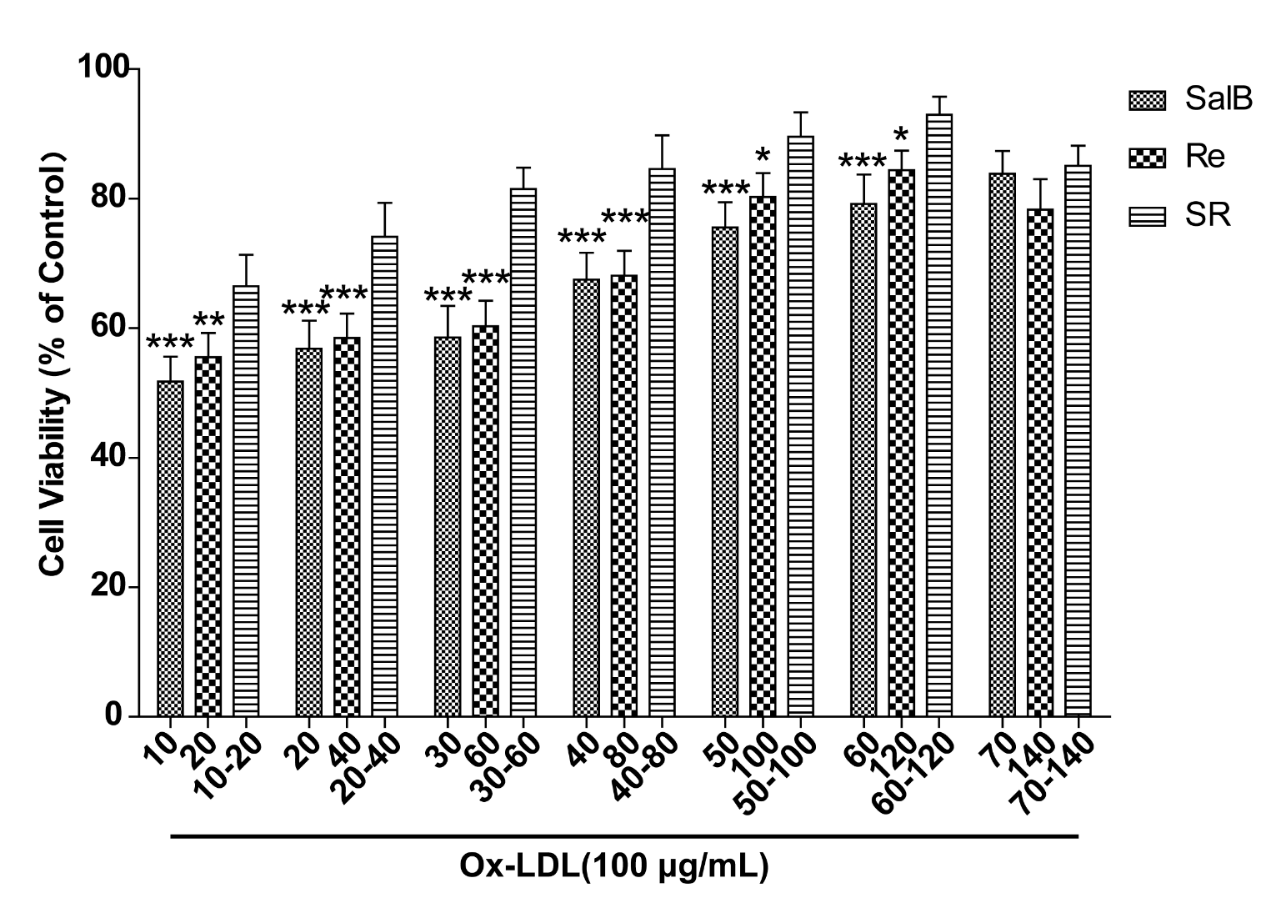


the cell viability with the combined treatment by MTT

Supplement: Supplementary file 1 [file Presentation_1.ZIP › supplemental material/supplemental material 5.docx]

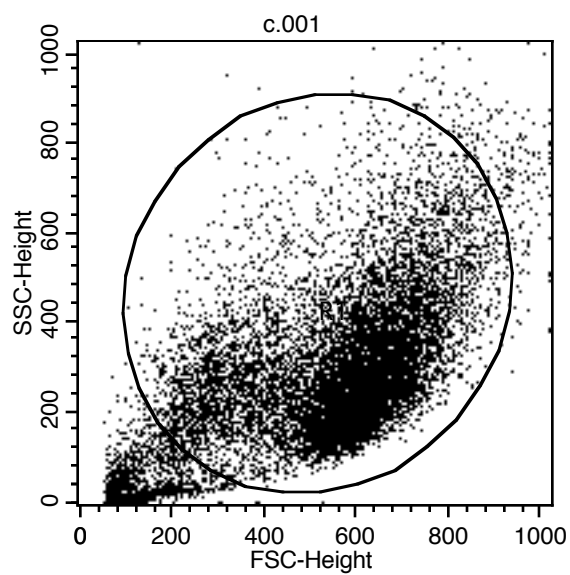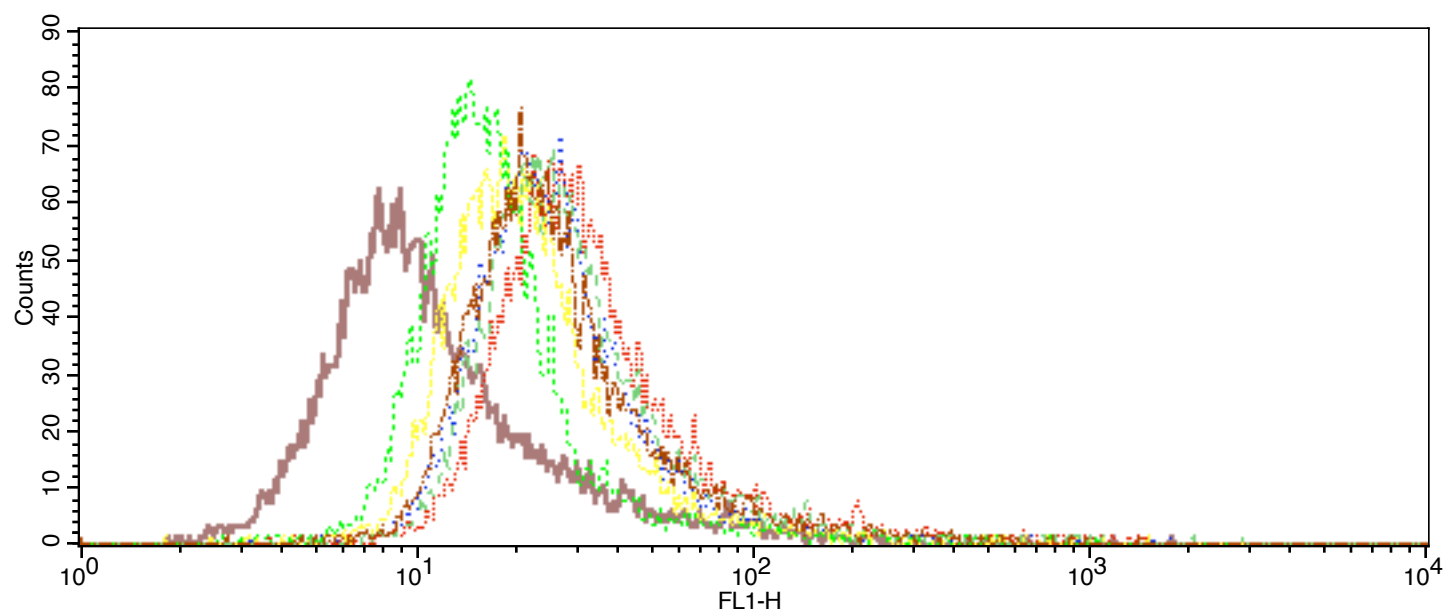

| Key     | Name        | Parameter | Gate |
|---------|-------------|-----------|------|
| —       | Control.003 | FL1-H     | G1   |
| .....   | Ox-LDL.002  | FL1-H     | G1   |
| ....    | SalB.005    | FL1-H     | G1   |
| ----    | Re.001      | FL1-H     | G1   |
| -----   | SR.005      | FL1-H     | G1   |
| - - - - | AM.002      | FL1-H     | G1   |
| - . - . | LY.003      | FL1-H     | G1   |

Supplement: Supplementary file 1 [file Presentation_1.ZIP › supplemental material/supplemental material 6/original image of cleaved-caspase3 by flow cytometry in paper.pdf]

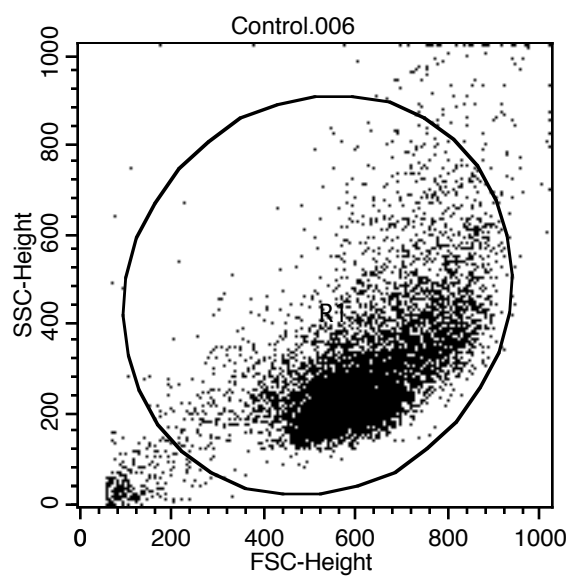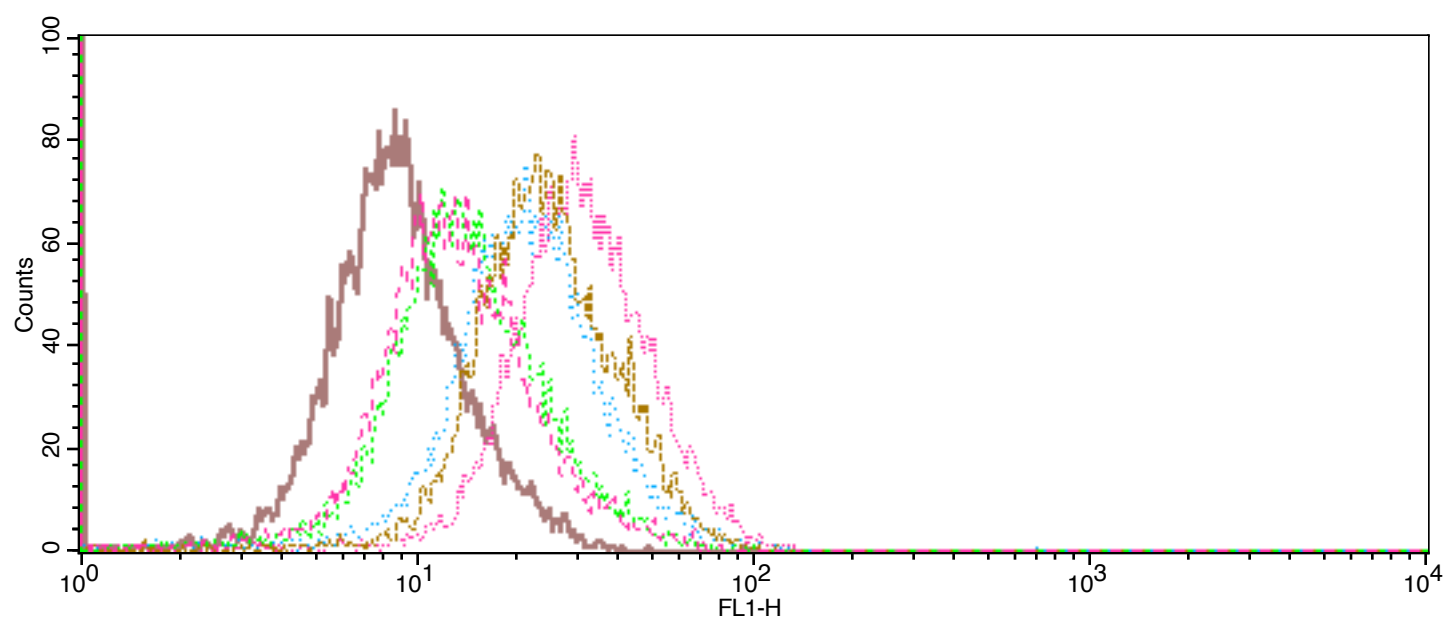

| Key   | Name        | Parameter | Gate |
|-------|-------------|-----------|------|
| —     | Control.006 | FL1-H     | G1   |
| ..... | Ox-LDL.001  | FL1-H     | G1   |
| ....  | SalB.002    | FL1-H     | G1   |
| ----  | Re.002      | FL1-H     | G1   |
| ----- | SR.002      | FL1-H     | G1   |
| - -   | NAC.002     | FL1-H     | G1   |

Supplement: Supplementary file 1 [file Presentation_1.ZIP › supplemental material/supplemental material 6/original image of ROS by flow cytometry in paper.pdf]

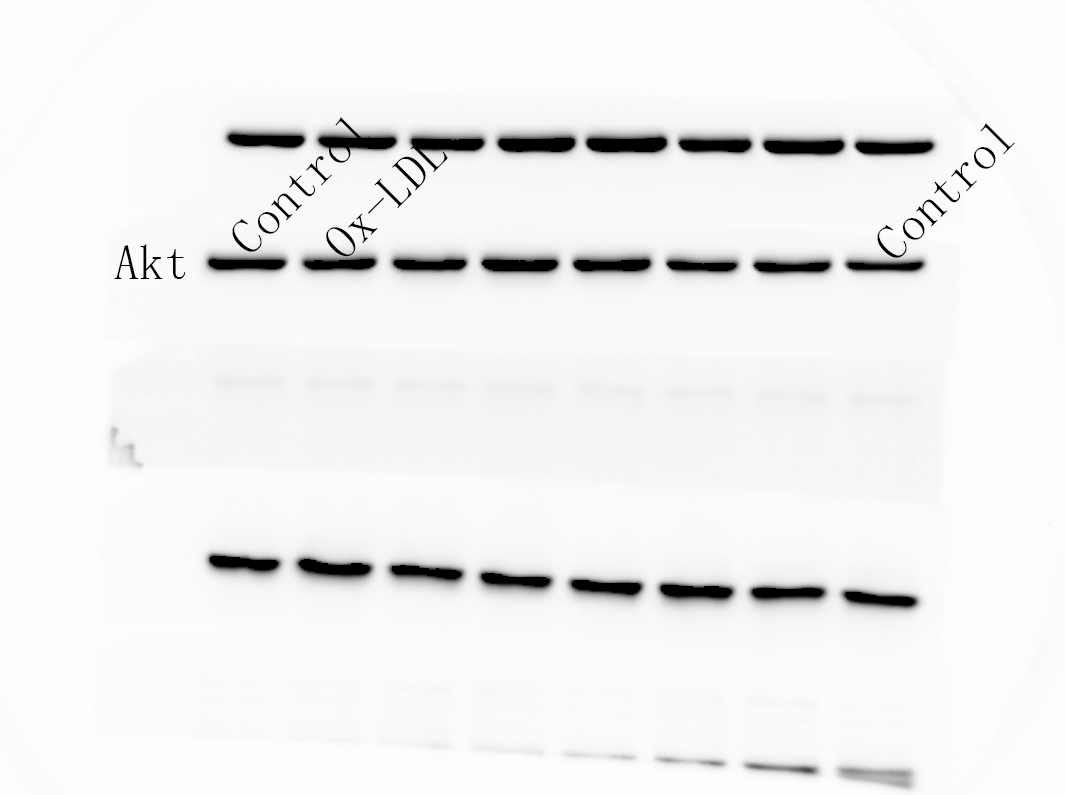

Supplement: Supplementary file 1 [file Presentation_1.ZIP › supplemental material/supplemental material 6/western blot/fig10/original image of Akt in paper.tif]

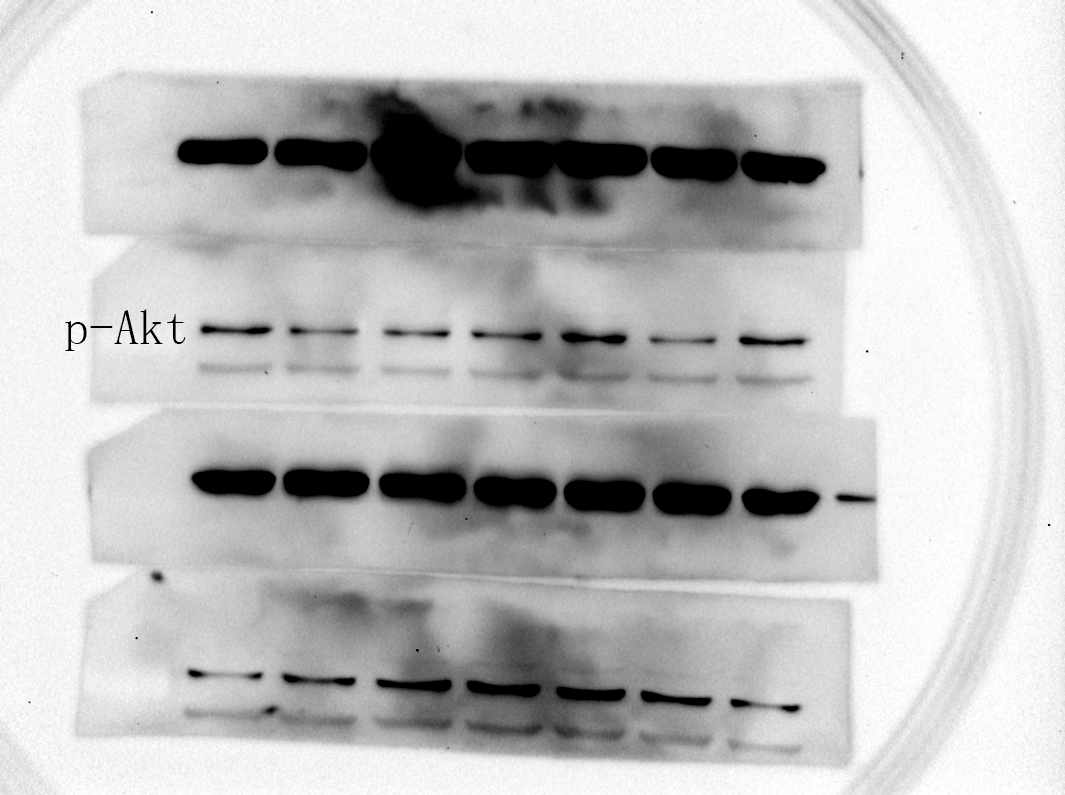

Supplement: Supplementary file 1 [file Presentation_1.ZIP › supplemental material/supplemental material 6/western blot/fig10/original image of p-Akt in paper.tif]

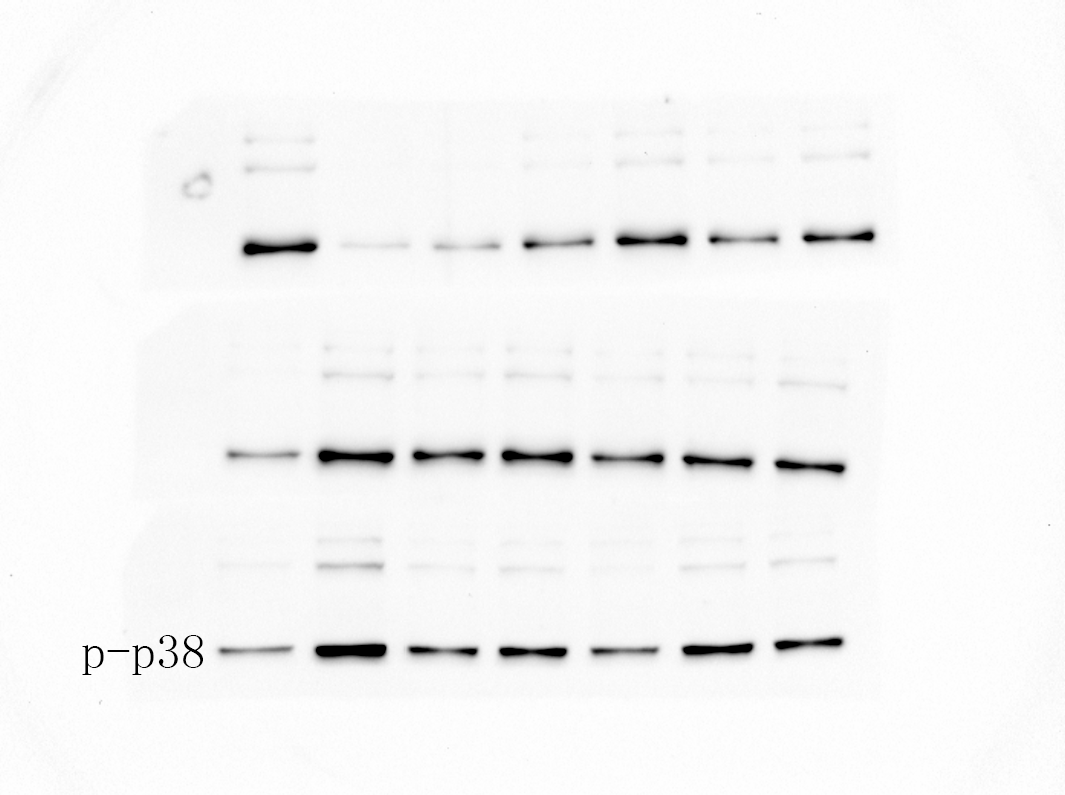

Supplement: Supplementary file 1 [file Presentation_1.ZIP › supplemental material/supplemental material 6/western blot/fig10/original image of p-p38 in paper.tif]

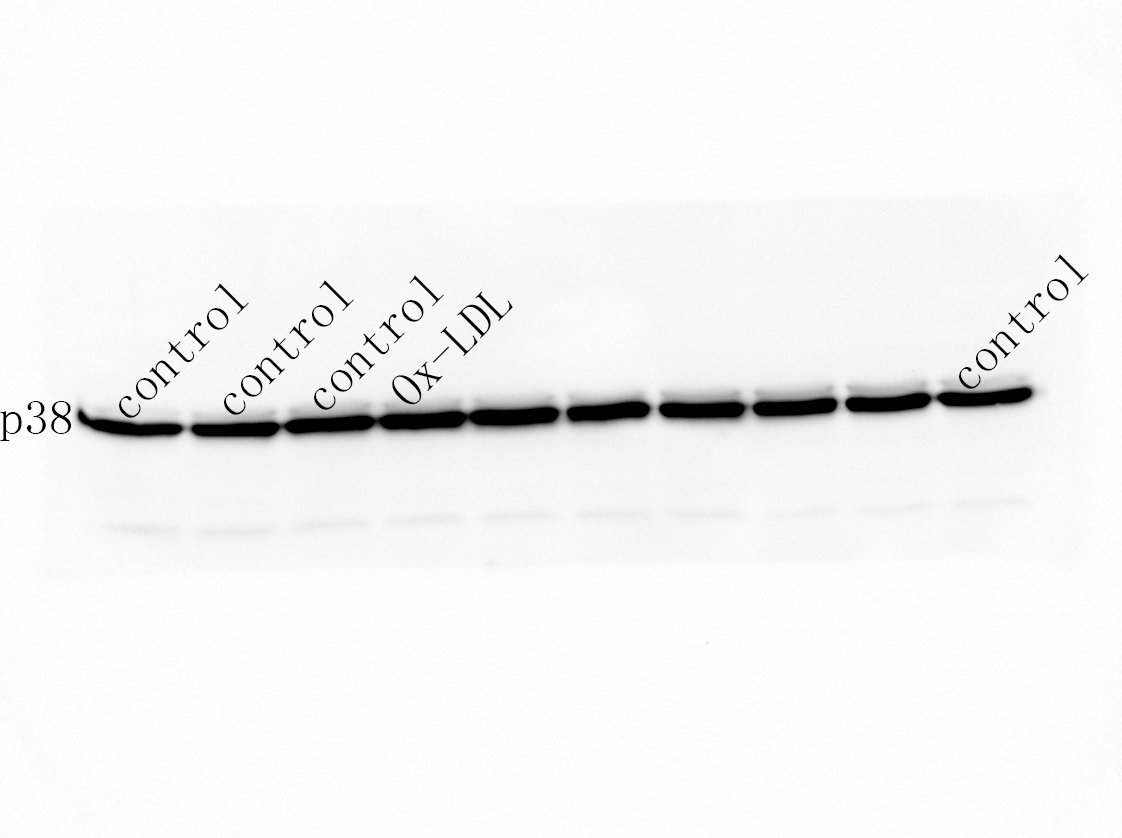

Supplement: Supplementary file 1 [file Presentation_1.ZIP › supplemental material/supplemental material 6/western blot/fig10/original image of p38 in paper.tif]

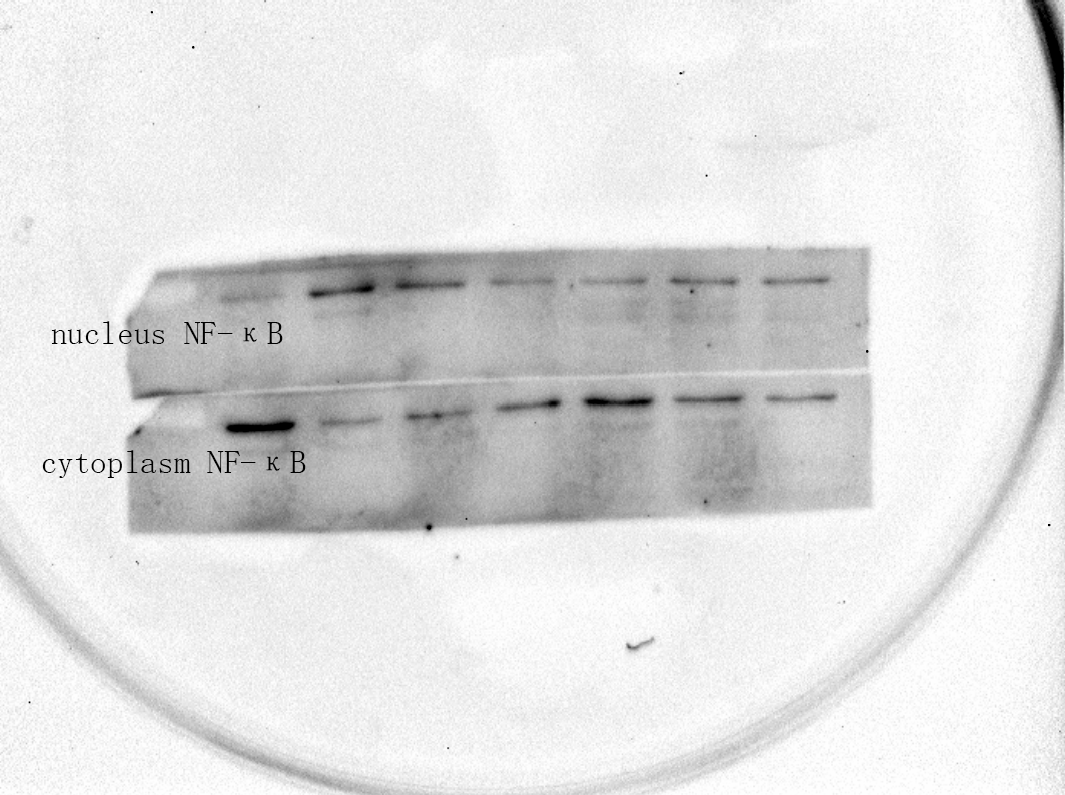

Supplement: Supplementary file 1 [file Presentation_1.ZIP › supplemental material/supplemental material 6/western blot/fig11/original image of nucleus and cytoplasm NF-κB in paper.tif]

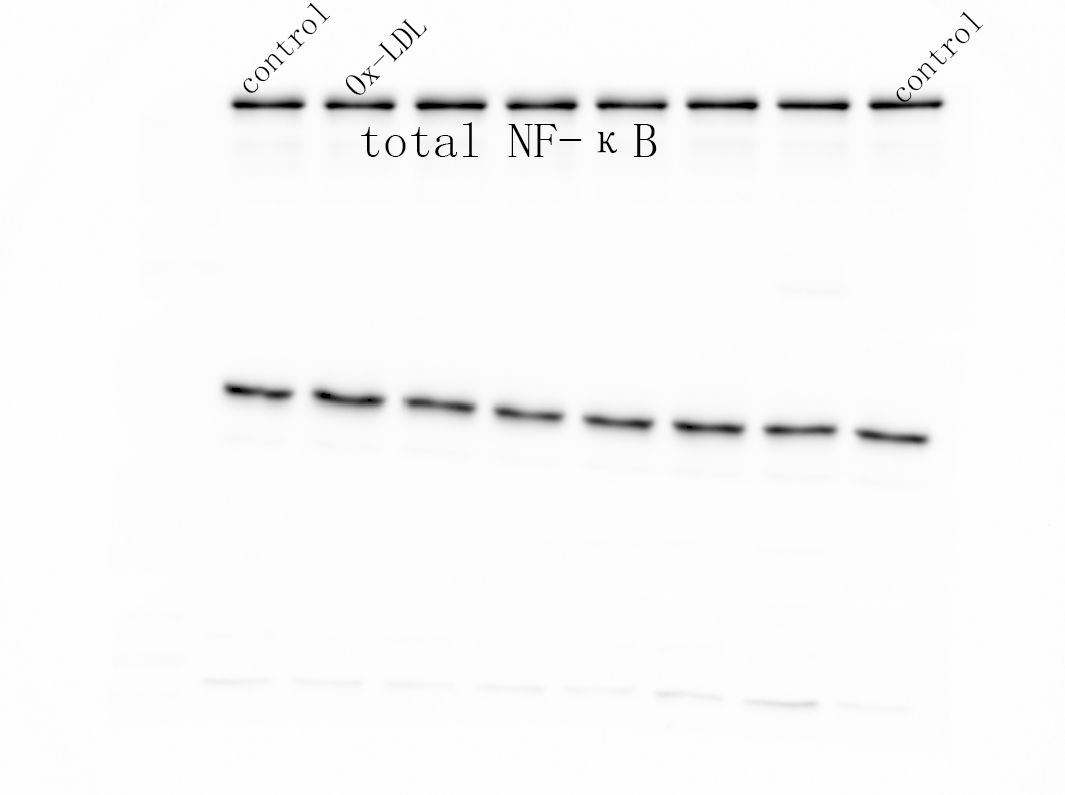

Supplement: Supplementary file 1 [file Presentation_1.ZIP › supplemental material/supplemental material 6/western blot/fig11/original image of total NF-κB in paper.tif]

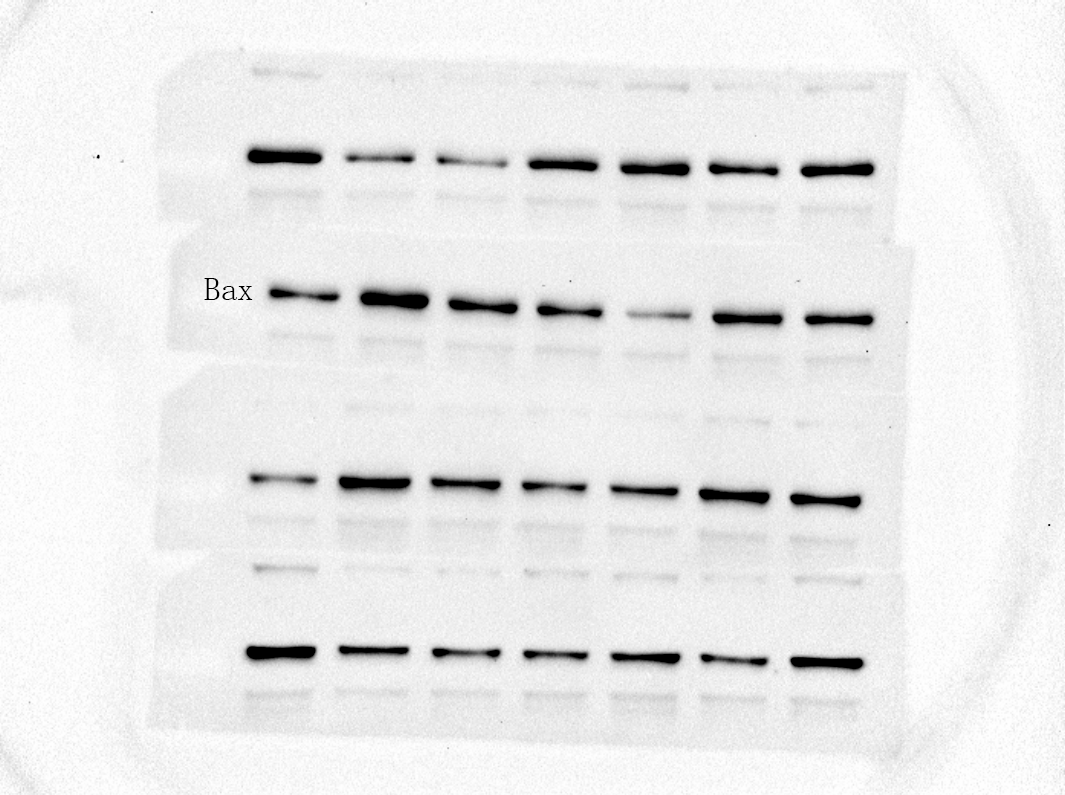

Supplement: Supplementary file 1 [file Presentation_1.ZIP › supplemental material/supplemental material 6/western blot/fig12/original image of Bax in paper.tif]

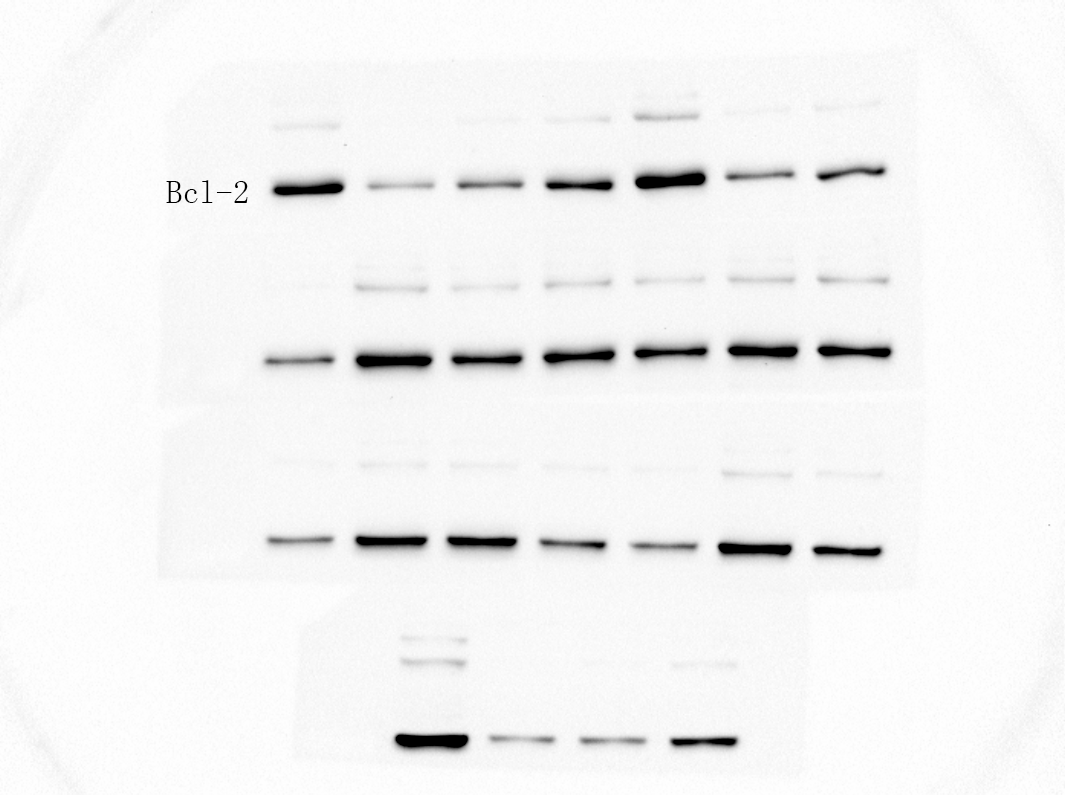

Supplement: Supplementary file 1 [file Presentation_1.ZIP › supplemental material/supplemental material 6/western blot/fig12/original image of Bcl-2 in paper.tif]

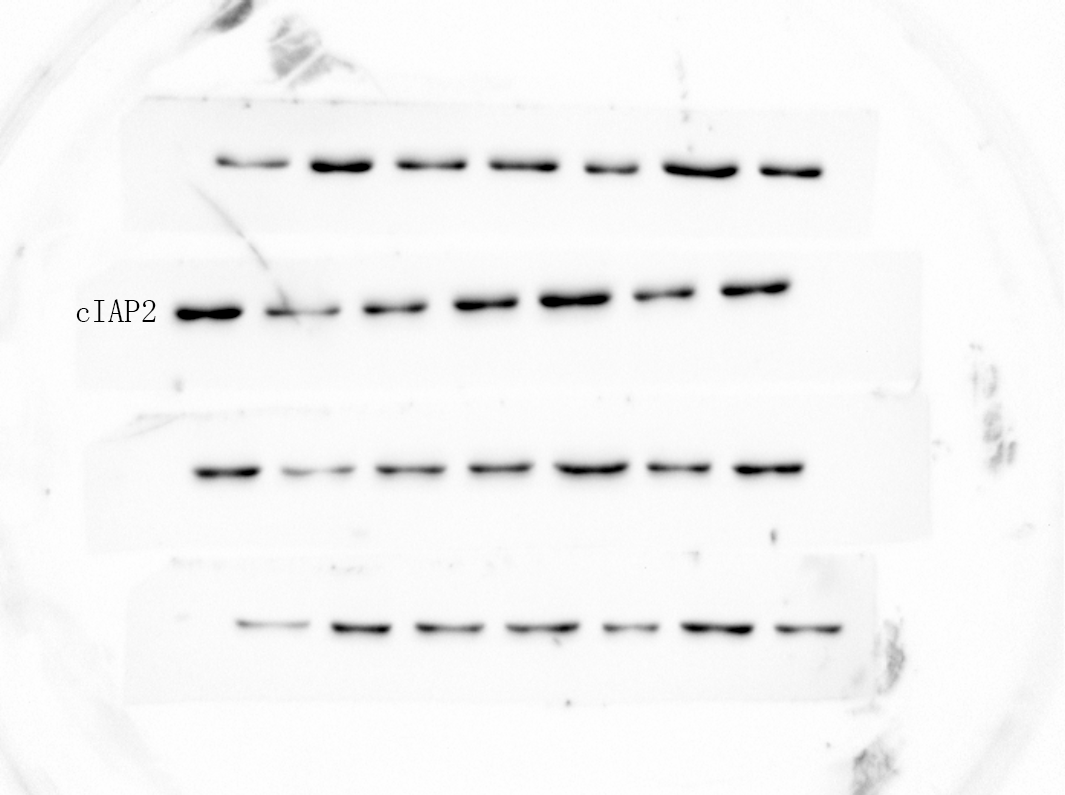

Supplement: Supplementary file 1 [file Presentation_1.ZIP › supplemental material/supplemental material 6/western blot/fig12/original image of cIAP2 in paper.tif]

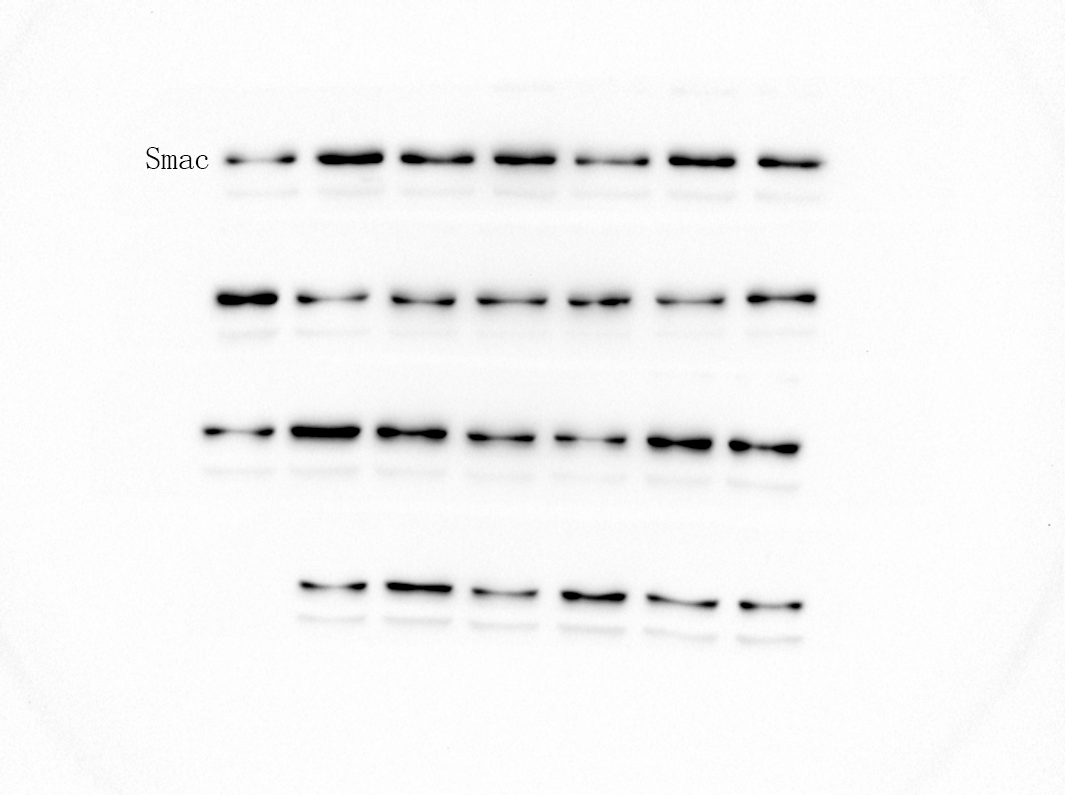

Supplement: Supplementary file 1 [file Presentation_1.ZIP › supplemental material/supplemental material 6/western blot/fig12/original image of Smac in paper.tif]

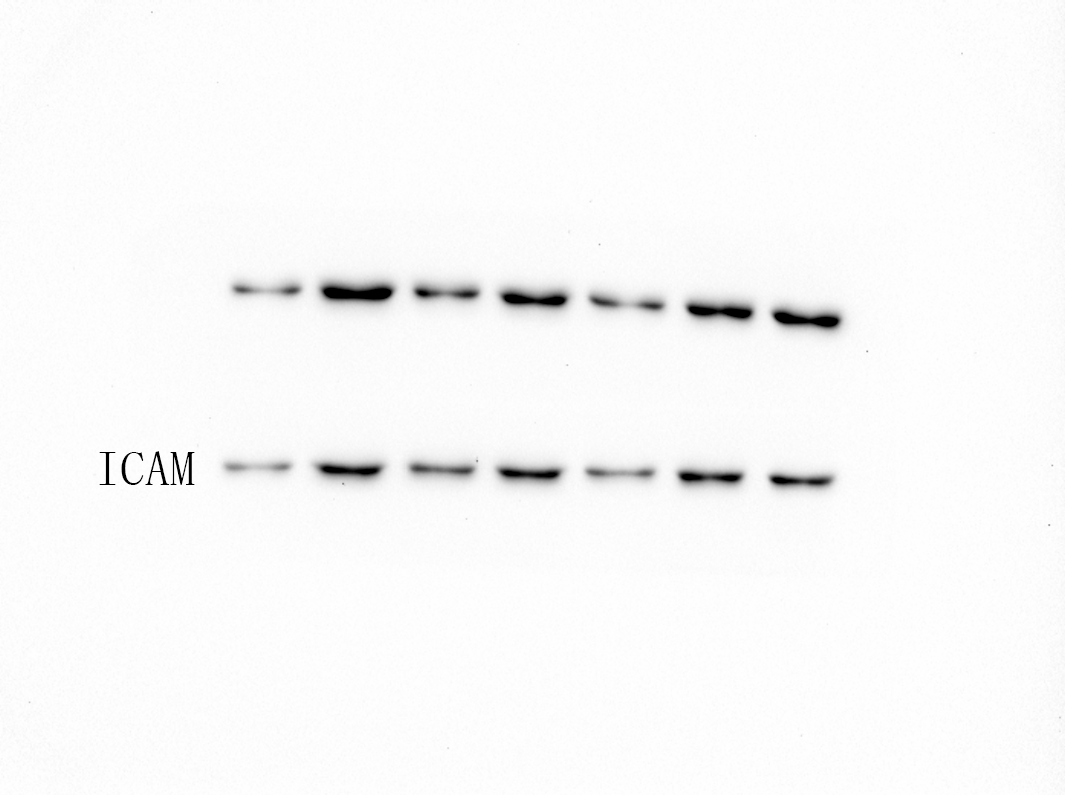

Supplement: Supplementary file 1 [file Presentation_1.ZIP › supplemental material/supplemental material 6/western blot/fig8/original image of icam in paper.tif]

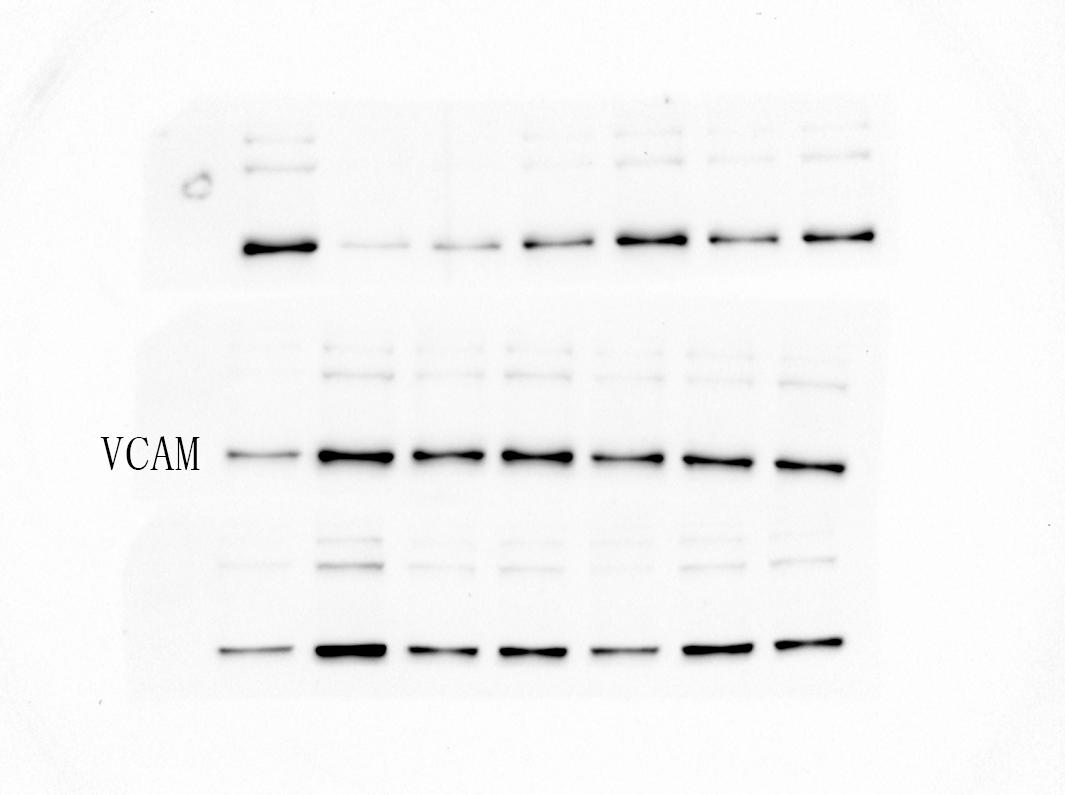

Supplement: Supplementary file 1 [file Presentation_1.ZIP › supplemental material/supplemental material 6/western blot/fig8/original image of vcam in paper.tif]

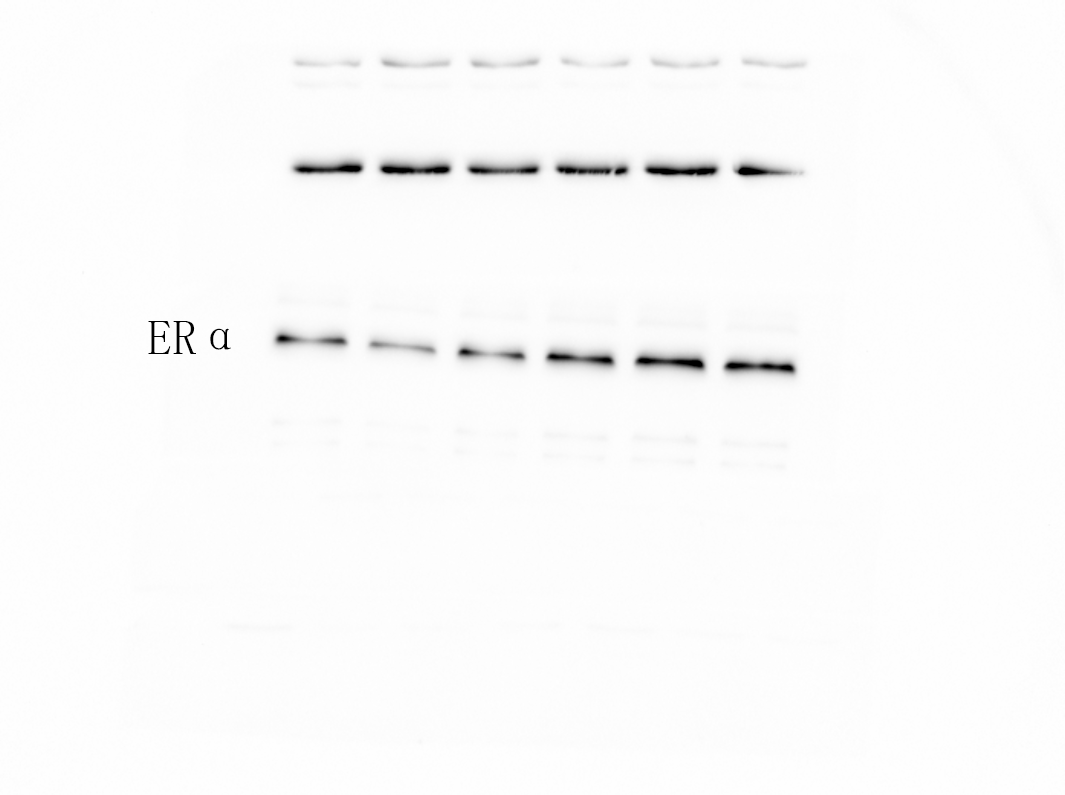

Supplement: Supplementary file 1 [file Presentation_1.ZIP › supplemental material/supplemental material 6/western blot/fig9/original image of ERα in paper.tif]

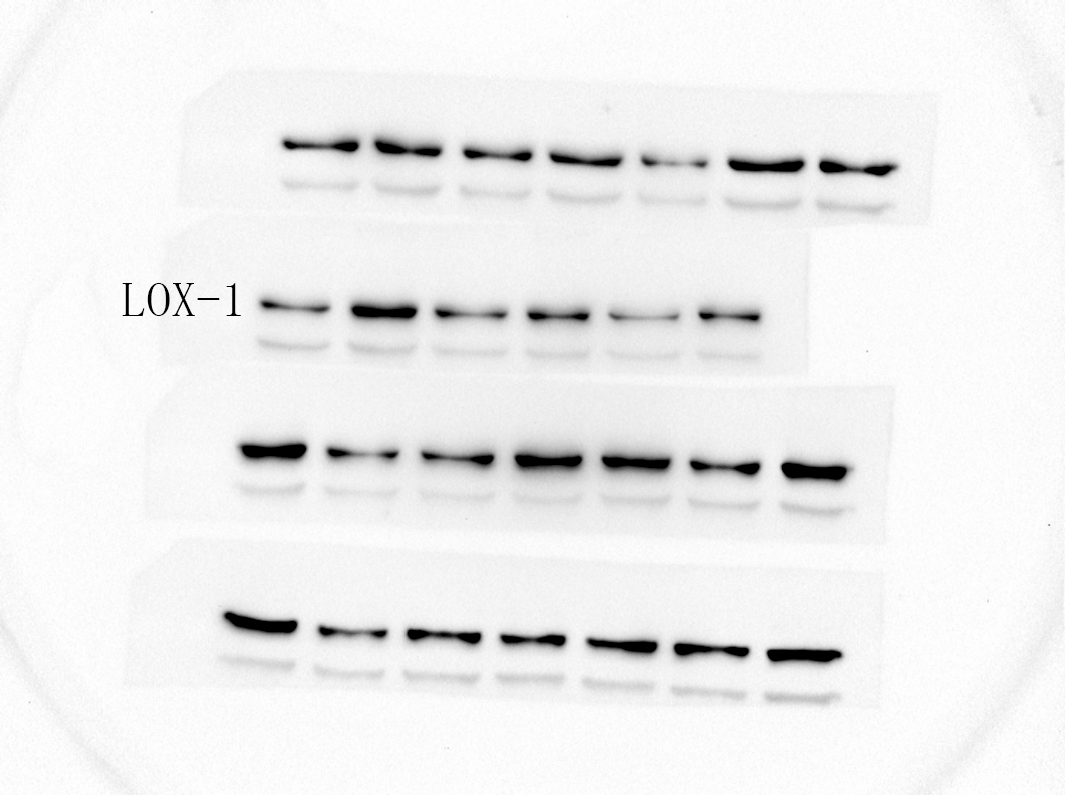

Supplement: Supplementary file 1 [file Presentation_1.ZIP › supplemental material/supplemental material 6/western blot/fig9/original image of LOX-1 in paper.tif]

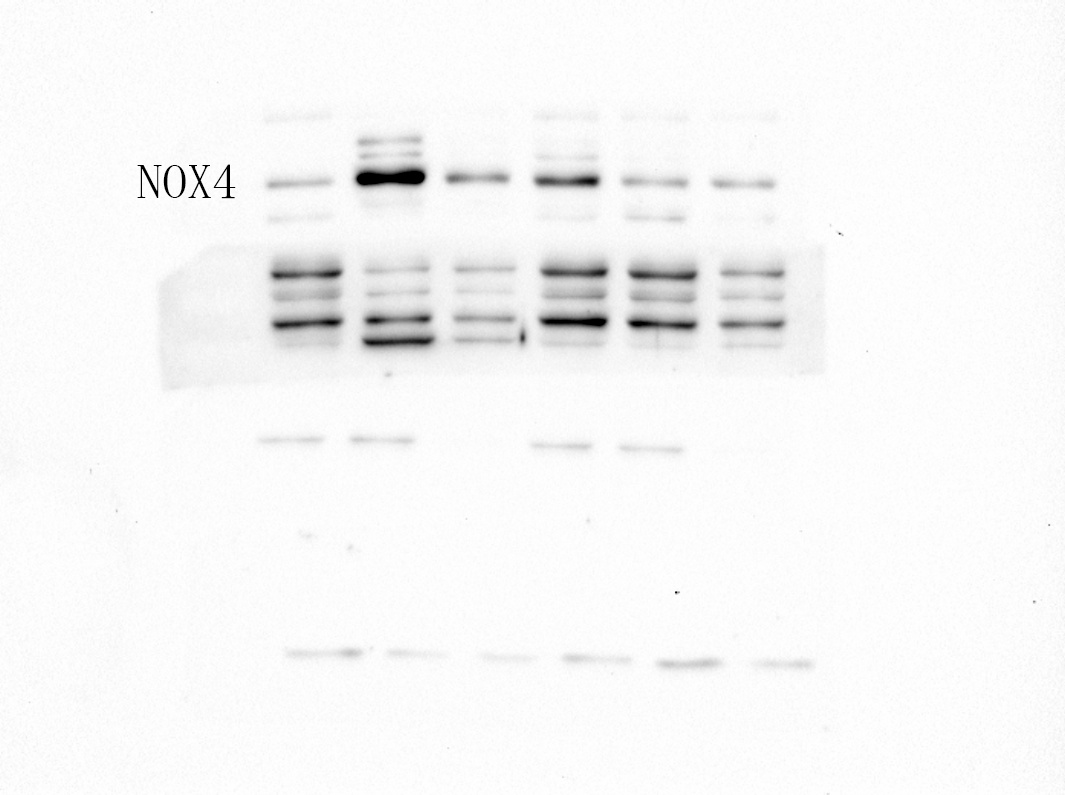

Supplement: Supplementary file 1 [file Presentation_1.ZIP › supplemental material/supplemental material 6/western blot/fig9/original image of NOX4 in paper.tif]
